# Format-Preserving Reduction of Canonical Nonlinear Models

**DOI:** 10.1007/s11538-026-01599-2

**Published:** 2026-03-04

**Authors:** Eberhard O. Voit

**Affiliations:** Department of Biological Sciences, University of Texas at Dallas, 800 W. Campbell Road, Richardson, TX 75080-3021 USA

**Keywords:** BST, Generalized mass action (GMA) system, Lotka–Volterra model, Nullcline, S-system

## Abstract

The recent upward trend in the size of mathematical models in the biomedical sciences offers novel opportunities and challenges. The latter are partially technical, for instance, in terms of computational efficiency and the need of vastly increased parameter determination, and partly conceptual, as large models make it more difficult to discern which variables are the key drivers of the model dynamics. The article proposes a model size reduction strategy that replaces differential equations with their corresponding nullclines. The result is an approximation whose quality depends on numerous aspects of the analyzed system. In the case of canonical S-systems and Lotka–Volterra models, the proposed reduction is essentially always feasible and retains their mathematical format, thereby facilitating sequential reductions. As these reductions are entirely formulaic, they are ideally suited for automation, which could systematically lead to models of optimally reduced sizes.

**Supplementary Information:**

The online version contains supplementary material available at 10.1007/s11538-026-01599-2.

## Introduction

Much of modern biology has developed into a computational science. Some computation has become necessary just to handle the abundance of data deriving from modern biomedical experiments, such as -omics and -multi-omics studies, where computational support plays a crucial role in data management and the analysis of significance and causality. Other areas of computation have become foci in their own right. These include bioinformatics, computational and structural biology, biomedical artificial intelligence and machine learning, network analysis, and dynamical systems analysis. This article focuses on the latter.

Dynamic analyses of biomedical systems are in most cases performed with systems of ordinary differential equations (ODEs), which dwarf—in their prevalence, although not in their importance—partial differential equation (PDE) models (e.g., Kuehn 2019), discrete models (e.g., Robeva 2015; Voit and Olivença 2022) and stochastic processes (e.g., Wilkinson 2019). ODEs have been used in biomathematical analyses for a long time, with models of the past containing relatively few variables, primarily due to insufficient data, but also due to difficulties determining parameter values from scarce data. This situation has been changing rapidly in recent times: current trends demonstrate strong increases in model size and complexity, sometimes containing dozens or hundreds of variables (Li et al. 2010; Snowden et al. 2017). This trend will continue unabatedly.

Models of larger size mandate the development of enhanced, effective methods for all steps of typical analyses. Some of these methods are of a technical nature, others are conceptual. Technical challenges pertain to algorithms for the analysis of large models, including the numerical integration of large systems of nonlinear ODEs. However, efficient solution algorithms are needed not only for simulations of large-scale systems of nonlinear ODEs, but ODEs must also be solved very many times in other instances, for instance, for the analysis of partial differential equation (PDE) models that are often assessed with approximations based on ODEs (Kuehn et al. 2019). Also, Monte-Carlo (MC) simulations have become a powerful and widely used tool for exploring the repertoire of possible, likely, and unlikely model responses (Harrison 2010; Velikova et al. 2024). In these simulations, the same model is solved thousands or even millions of times with parameter values obtained from some type of randomization (Liu 2018). Importantly, the benefits of MC simulations in terms of insights and understanding increase with the size and complexity of the analyzed model. Finally, the estimation of parameter values of nonlinear ODE models essentially always requires thousands of repeated solutions of these models with adjusted parameter values; for a rare exception, see Voit and Almeida (2004).

In terms of conceptual challenges, it is not always a priori clear which variables in large systems are the true drivers of their dynamics and which are more or less necessary bystanders. Without clear knowledge of a variable's importance, it is challenging to design and implement large models in an effective manner.

In an effort to address these questions, this article discusses reductions of the size ("order") of canonical ODE systems. It supposes that the modeler is particularly interested in one or a moderate number of true drivers of system dynamics, while other variables may be biologically relevant but do not affect the dynamics of the system much. Snowden et al. (2017) calls the size reduction of ODE systems a “vital topic” and Padoan et al. (2021) consider the topic of approximating the global behavior of nonlinear systems with smaller systems as important but “largely open.”

The reduction of complexity to "representative simplicity" is a key topic in the important search for emergent behaviors (Lorenz et al. 2011; Mossio et al. 2013) and for motifs and design and operating principles in biomedical systems (Alon and An 2019; Milo et al. 2002; Savageau 1985, 1989). Beyond a deeper understanding of these systems, recognizing such principles is a prerequisite for targeted interventions and manipulations of biomedical systems, for instance, in synthetic biology, quantitative systems pharmacology, and metabolic engineering. A plethora of examples, including (Alves and Savageau 2000a, b; Andrews et al. 2023; Bromig et al. 2020; Irvine and Savageau 1985; Lee et al. 2011; Ma et al. 2009; Ma'ayan et al. 2008; Poyatos 2012; Savageau 1998; Savageau et al. 2009; Stone et al. 2019; Voit 2004), makes it evident that the search for design and operating principles is essentially always performed on "stripped-down" versions of realistically detailed systems.

The size reduction of ODE systems raises obvious questions of validity. For instance, is it acceptable to ignore intermediates of a metabolic pathway. Clearly, from a biochemical standpoint, intermediates are necessary because a substrate cannot be directly converted into a downstream product. Also, they tend to create a slight time delay, but are they otherwise needed or of interest? Are the delays really important? Is their dynamics of pertinence for insights into the functioning of the system? More generically, one must ask: (1) Can reductions help categorize variables and identify the true drivers of the dynamics of a system? (2) Is it possible to retain the original modeling format while reducing the number of variables?

The ideas behind the reduction method proposed here are similar to proposals by Michaelis and Menten (1913), Briggs and Haldane (1925), Klonowski (1983) and Tikhonov (1952), and many others (see Sect. 2). However, there are two distinctly different aspects. First, the most closely related methods assume noticeably different time scales among the system variables, to a point where the left-hand sides of some ODEs are assumed to converge to zero. By contrast, different time scales are not assumed here. Instead, setting some derivatives equal to zero is considered an approximation that may or may not capture the key features of the system under the given conditions to an acceptable degree. Expressed differently, each reduced equation represents a nullcline, as it is used in phase-plane analysis (Strogatz et al. 2024; Voit et al. 2025). Secondly, the methods proposed here preserve the original canonical modeling format, which is important for successive reductions, the identification of the drivers of system dynamics, and the eventual automation of the process. Finally, the results of the reductions are again first-order ODEs, rather than higher-order systems of lower dimensionality (Harrington and Van Gorder 2017).

## Background

### Model Reduction Methods

At first glance, the number of variables in a model seems to be clear-cut and beyond questioning. However, numerous factors can increase or decrease this number (Voit 1992a). As two opposite examples, Lie-group transformations (Olver 1986; Voit 1992b) and methods of differential algebra (Harrington and Van Gorder 2017) can reduce the number of ODEs, whereas the method of recasting (Hernandez Bermejo and Fairen 1997; Peschel et al. 1986; Savageau and Voit 1987; Voit and Savageau 1986) converts arbitrary ODE models into canonical models through the introduction of auxiliary variables.

Many existing model reduction methods have the benefit that fewer parameters must be estimated. Expressed from a different viewpoint, the original model may be too complex for the available experimental data, thus suggesting the search for a model that is clearly simpler than reality but commensurate with the data.

Due to its potentially great benefits, model reduction has been approached for a long time and in numerous ways. Many of these methods are quite complex and difficult to use in practical applications, and some apply only to linear systems. Surveys and reviews include (e.g., Antoulas 2005; Baur et al. 2014; Benner et al. 2017; Besselink et al. 2013; Brenner et al. 2013; Gorban et al. 2011; Lu 2021; Løvås 2012; Radulescu et al. 2012; Rosmalen et al. 2021; Padoan et al. 2021; Snowden et al. 2017; Vora and Daoutidis 2001). Categorized in broad strokes, the most prevalent model reduction approaches involve: (1) separation of time scales by approximating particularly fast and/or slow processes as constant (Gerdtzen et al. 2004; Kourdis et al. 2013; Krüger and Heinrich 2004; Lee and Othmer 2010); (2) exploitation of system modularity by clustering and lumping variables into modules (Anderson et al. 2011; Danø et al. 2006; Huang et al. 2010; Liao and Lightfoot 1988; Wei and Kuo 1969); (3) sensitivity analysis to remove insensitive parameters or variables (Degenring et al. 2004; Huang et al. 2010; Liu 2018; Quaiser et al. 2011; Smets et al. 2002; Tomlin and Ziehn 2011; Transtrum and Qiu 2014, 2016); and (4) balanced truncation for reducing linearized systems via reachability and observability analysis (Hahn and Edgar 2002; Liebermeister et al. 2005; López-Caamal and Marquez-Lago 2014; Moore 1981; Snowden et al. 2017). Yet other approaches explore the conformal symmetry or scale invariance using renormalization group techniques (Goldenfeld 1992; Zinn-Justin 2007). Furthermore, in rare cases, equations may be decoupled from the system through Lie-group transformations (Mobeen Munir et al. 2023; Olver 1986; Voit 1992b). Differential elimination (Harrington and Van Gorder 2017), based on sophisticated differential algebra, can reduce the model dimension, sometimes even to a single equation, which however is typically of higher order.

#### Time-Scale Separation

A well-known early example for time-scale separation is the derivation of the Michaelis–Menten rate law (Briggs and Haldane 1925; Flach and Schnell 2006; Heineken et al. 1967; Michaelis and Menten 1913). In a nutshell, the enzymatic conversion of a biochemical substrate into a product occurs in two steps, namely the reversible formation of a complex between substrate and enzyme, and the irreversible disassembly of this complex into product and reusable enzyme. The mechanism was originally modeled as a mass action system, which consisted of three ODEs that at the time were difficult to handle. The proposed solution was to consider the concentration of the complex as constant, which permitted the rate of product formation to be formulated as an explicit function of the substrate concentration. In much more general terms, Tikhonov and others (Klonowski 1983; Snowden et al. 2017; Tikhonov 1952) considered systems with variables operating at vastly different time scales and considered cases where the differentials on the left-hand sides of some ODEs can be assumed to converge to zero. The resulting algebraic equation is then used to replace the ODE. In opportune cases, the algebraic equation can be explicitly solved for the reduced variable and substituted back into the remaining ODEs, but that is not always the case.

#### Modularity

Many physiological and biochemical systems can be partitioned into modules that act as interacting autonomous modules (Alcalá-Corona et al. 2021; Hartwell et al. 1999; Hatleberg and Hinman 2021; Kadelka et al. 2023; Lauffenburger 2000), suggesting that representative computational models might be structured in analogous ways (Kumbale et al. 2025, 2021; Schnell et al. 2007) and that they could provide guidance for reduction strategies (Snowden et al. 2017).

#### Methods Based on Sensitivity Analysis and Optimization

These approaches attempt to optimize aspects of the system functionality within an acceptable range of inaccuracy. Generic approaches either use the sensitivity of a variable to parametric or structural changes as a guide for model reduction or employ trial and error to decide among many alternative reductions (Perumal et al. 2009; Snowden et al. 2017). Model reduction per sensitivity analysis can be combined with "manifold learning": Given a set of experimentally observed time series data and a complex model, a model is viewed as a high-dimensional geometric manifold and the data collectively as a point on (or close to) this manifold. Sensitivity analysis is then used to identify the manifold boundary closest to the data point, which is a sub-manifold that encodes the most appropriate reduced model, which typically contains fewer parameters (Transtrum and Qiu 2014). This manifold approach is applicable to any continuous and differentiable model formulation, while its limitation is the focus on a given set of experimental data, which only provides insights into the mechanism specific to these data.

These and other reduction methods offer partial solutions. First, they are only applicable to certain cases, such as dynamical systems with well-separated time scales or modular components. Second, these methods are typically applied based on ad hoc approximations that require expert intuition, but have little rigorous justification and hence are difficult to implement in a systematic—or automated—manner. Third, reduced models produced by these methods are often disconnected from the original models, because parameters in the reduced models often do not explicitly match the parameters in the original model; a well-known analogy of this mismatch is model-dimensionality reduction using Principal Component Analysis (Duda et al. 2001; Snowden et al. 2017). Finally, although the simplified models produced by these methods are able to preserve an experimentally observed behavior, they offer little insight regarding the mechanisms that give rise to this observed behavior of the original model.

### Canonical Modeling Formats

The design of a model has traditionally been the core activity of the biomathematical modeler. It starts with the collection of pertinent information, the omission of information deemed to be of secondary importance, the abstraction of complex components through simplifying assumptions, and the arrangement of all retained pieces of information into a diagram (Voit et al. 2025). For increasingly larger models, this model design step at first only becomes cumbersome, but it can ultimately be overwhelming, if not infeasible. Promising help, not yet materialized, will likely come from the targeted employment of machine learning and artificial intelligence (AI).

The translation of the diagram into a functional model is arguably the least-well defined, because there is no nature-given guidance regarding the format of these functions that is truly defensible and objective. Physics is governed by laws faithfully describing forces, electrical or optical processes and other phenomena. Biology must obviously obey these laws, but biomedical phenomena are almost always so convoluted that a true biophysical representation is infeasible (Voit 2017; Voit et al. 2010).

A potent approach to overcoming this lack of natural guidance is the use of *canonical models*, which are set up according to rigorously prescribed rules. The best-known canonical examples are power-law models in Biochemical Systems Theory (BST), which include mass action systems as special cases (see below), and Lotka–Volterra (LV) models. Canonical models offer two crucial advantages. First, they unambiguously prescribe how to convert diagrams into equations, which is a fundamental prerequisite for automatization, for instance, with methods of machine learning and AI. Second, each canonical format permits a one-to-one mapping between the diagram and the model equations, with very few exceptions of no particular importance (see next sections). The consequence is that the model equations identify directly which variables are involved with which particular processes.

#### Power-Law Models (GMA and S-Systems)

**Fig. 1:**
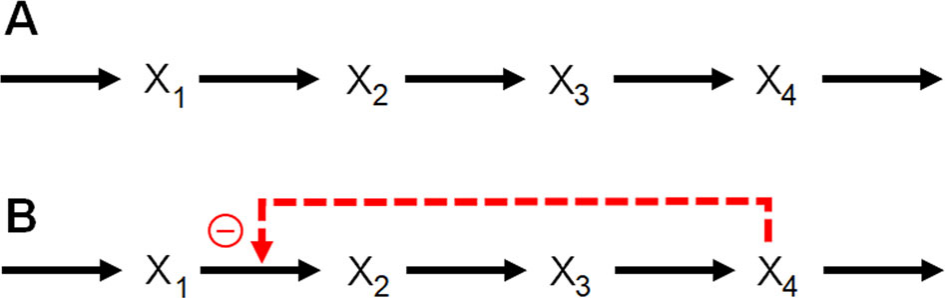
Two similar pathways, one with internal feedback and one without.

The power-law approximation (PLA) of all processes in a system is the foundation of Biochemical Systems Theory (BST; Savageau 1969a, 1976; Voit 2000, 2013). It is based on Taylor linearization in logarithmic space, which is equivalent to a nonlinear representation in Cartesian space. Specifically, every process is represented as a product of a rate constant and of all variables directly affecting this process, raised to an exponent called a kinetic order. As an illustration, consider the power-law representation of the conversion of *X*_1_ into *X*_2_ in the two situations of Fig. 1. In case **A**, the production of *X*_2_ depends exclusively on *X*_1_ and is formulated in BST as $\gamma {X}_{1}^{{f}_{1}}$. In case **B**, this process also depends on the inhibitor *X*_4_ and is therefore formulated as $\gamma {X}_{1}^{{f}_{1}}{X}_{4}^{{f}_{2}}$. The parameter *f*_2_ reflects the inhibition and is therefore negative, with a magnitude corresponding to the strength of the inhibition: If this inhibition is decreased in strength, *f*_2_ decreases in magnitude and becomes 0 once the inhibition ceases to be present. In this case, diagram **B** simplifies to **A** and $\gamma {X}_{1}^{{f}_{1}}{X}_{4}^{{f}_{2}}$ becomes $\gamma {X}_{1}^{{f}_{1}}$.

The offshoot of his one-to-one mapping between the diagram and the model equations is that unimportant variables disappear from the equations (almost or entirely), by having associated exponents close to zero, while the strong drivers of the system dynamics have positive or negative exponents substantially different from zero.

PLA is a local approximation, which entails that it is exact at an operating point of choice, very good close to this point, and of a quality that is difficult to determine for points further away from the operating point. Because many biomedical systems operate relatively close to a nominal steady state, often referred to as homeostasis, the dynamics of a PLA model often does not differ much from more traditional formats (e.g., Alvarez-Vasquez et al. 2004; Curto et al. 1995; Sorribas and Savageau 1989; Voit and Savageau 1987) and is surprisingly good in practical applications, although exceptions can of course be constructed (e.g., Heijnen 2005; Wang et al. 2007).

PLA permits variant strategies, the best known of which are Generalized Mass Action (GMA) systems and S-systems. In the GMA format, every process is represented with its own power-law term, which aligns well with intuition. The result is

1 $${\dot{X}}_{i}={\sum }_{k=1}^{{T}_{i}}\pm {\gamma }_{ik}{\prod }_{j=1}^{n+m}{X}_{j}^{{f}_{ikj}}, i= 1, \dots , n$$

where *T*_*i*_ is the number of terms in the *i*^th^ equation, the variables *X*_1_, …, *X*_*n*_ are dependent (state) variables, and the variables *X*_*n*+1_, …, *X*_*n*+*m*_ are independent variables that affect the system but are not affected by the system.

In the S-system format, all fluxes entering a given system component are collectively represented with a single power-law term and the same is done with all fluxes leaving this component. As a consequence, an S-system never contains more than two terms with opposite signs:

2 $$\stackrel{ .}{{X}_{i}}={\alpha }_{i}{\prod }_{j=1}^{n+m}{X}_{j}^{{g}_{ij}}-{\beta }_{i}{\prod }_{j=1}^{n+m}{X}_{j}^{{h}_{ij}} \quad i = 1, \dots , n.$$

The advantage of this S-system format is that the steady-states of arbitrarily large systems and many features associated with them can be assessed analytically (Savageau 1969b; Voit 2000). While the format may seem very restrictive, S-systems have been employed in a variety of applications, such as metabolism (Savageau 1969a, b, 1976; Voit 2013, 1991; Torres et al. 2002), metabolic engineering (Brown 1991; Garcia and Torres 2011; Torres et al. 2002), analyses of energy production (Horner and Wolinsky 2002; Jorquera et al. 2008; Zhang 2011), chemical network theory (Arceo et al. 2015; Papachristodoulou and Recht 2007; Searson et al. 2007), general systems theory (Antoniotti et al. 2002; Lewis and Voit 1991; Peschel et al. 1989; Streichert et al. 2004; Voit and Veflingstad 2008), numerical analysis (Burns and Mueller 1999; Irvine and Savageau 1990; Savageau 1993; Shiraishi and Hatoh 2007), and computational statistics (Chou et al. 2000; He and Voit 2005; Rust and Voit 1990; Voit and Knapp 1997). Intriguingly, this format is very rich and, with auxiliary variables, can represent any continuous nonlinearities exactly (Savageau and Voit 1987; Voit and Savageau 1986). The design of power-law models from a diagram is unique, except for the choice between GMA and S-system formats. Similarly, the interpretation of power-law equations in terms of a diagram is unique, except for trivial nuances, such as a variable affecting its own degradation in different, simultaneous ways, such as being the substrate of the degradation process and also inhibiting it.

#### Lotka–Volterra (LV) Models

LV models represent the logarithmic change in each variable over time with a linear function of all variables directly affecting this variable. In Cartesian coordinates, the result is

3 $${\dot{X}}_{i}={a}_{i}{X}_{i}+{\sum }_{j=1}^{n}{b}_{ij}{{X}_{i}X}_{j}, i = 1, \dots , n$$

All parameters may be positive, negative or zero.

LV models have traditionally been used to model interactions among coexisting populations (Dakos et al. 2020; Dam et al. 2020; Davis et al. 2022; Dimas Martins and Gjini 2020; Lotka 1920; May 1973; Olivença et al. 2022; Volterra 1926; Wangersky 1978). However, they have also been used in a variety of other applications, for instance in physics (Hacinliyan et al. 2010; Nambu 1986), economics (Chiang 2012; Vadasz et al. 2017; Zhou and Chen 2006), conflict analysis (Orlando et al. 2021), marketing (Orbach 2022), pollution analysis (Haas 1981), and to improve ocean productivity through iron fertilization (Pan et al. 2015). Finally, it has been shown with rigorous mathematical analysis that the LV model structure, like the power-law structure, is extraordinarily rich. Indeed, it is capable of capturing any differentiable nonlinearities, including stable limit cycle oscillations and deterministic chaos (Sprott et al. 2005; Vano et al. 2006), if sufficiently many auxiliary variables in LV format are introduced (Hernandez Bermejo and Fairen 1997; Peschel et al. 1986; Voit and Savageau 1986).

### Reduction of Canonical Models

An interesting opportunity hovers at the intersection of canonical models and model reduction, namely, the practical reduction of larger systems toward key system nodes and mechanisms, while retaining the canonical modeling format.

The approach proposed here supposes that a canonical system model is known with all its specifications and explores whether one can construct a simpler canonical model that has essentially the same dynamics. This type of reduction is beneficial for simplifying the analysis and becomes increasingly more critical with larger model sizes (Li et al. 2010; Snowden et al. 2017). Maybe more important, biological modelers frequently encounter the situation that only features associated with key variables had been measured experimentally, whereas there is little information on secondary components of a system; this typical scenario creates obvious issues for parameter estimation. The reduction method proposed here ideally allows the reduction of complex models to key variables and offers a tool for evaluating to what degree some of the system variables are truly of secondary importance. If they are, parameter estimation can be executed with a (much) smaller and better identifiable model (e.g., Srinath and Gunawan 2010), which in the process directly demonstrates dependencies among parameters. Furthermore, all parameters still have a directly interpretable meaning.

It is clear that the reducibility of models depends on a multitude of factors, including: the model topology and regulation; the chosen output variables that are to be retained; parameter values; as well as types and magnitudes of perturbations considered tolerable. The ideal solution would be a “theory of reducibility,” but the complicated nature of the problem is presently obscuring what such a theory might be. Two Results sections speculate on potential approaches toward a theoretical basis of the heuristic reduction.

## Methods

### General Requirements and Assumptions

The proposed method replaces ODEs in a system with their corresponding nullclines, that is, with the derivative set equal to 0. This replacement is naturally best close to a (stable) steady state, and because natural systems typically operate close to homeostasis, the reduction is expected to work rather well in practical applications. Some natural systems exhibit limit cycles or chaotic oscillations, in which case the pertinent steady states are unstable. The reduction is typically problematic in these cases, but a later example will demonstrate that the limit-cycle behavior is sometimes retained nevertheless. Bistability is retained in the reduction, which contrasts typical time-scale separation schemes (Flach and Schnell 2006; Padoan et al. 2021).

One advantage of prioritizing S-systems and LV systems is that nullcine equations can almost always be solved for the variable of interest. Rare exceptions will be discussed later. This solvability is unusual for other model types, including GMA models. Nonetheless, as far as the nullcline equation can be solved, a reduction is possible in GMA and non-canonical models. An example will demonstrate this aspect.

It is important to keep in mind that the reduction is an approximation. As with every approximation, the method works well close to an operating point, but may or may not work well far away from this regime. Generically, the approximation quality depends on: the required accuracy throughout the time courses of the system variables; the effect of a variable on the main output(s) of interest; and the relaxation time of a variable. Typically, model reduction shortens or eliminates time delays caused by intermediate variables. If these are critical, the reduction is often unduly inaccurate. In some cases, a scaling of time can remedy this shortening of time delays. The example of a molecular cascade will demonstrate this scaling.

In the following, the variables of all original systems are called *X*_*i*_, while the corresponding variables of the reduced systems are called *Y*_*i*_.

### Systematic Reducibility in Canonical Models

We begin the discussion with S-systems, where the methodology is clear-cut, followed by GMA systems, and then Lotka–Volterra systems. Detailed examples for all three are presented in the Sect. 4.

#### Reduction of S-Systems

Suppose, the task is to reduce the *k*^th^ ODE of an S-system (see Eq. 2) that possesses a non-trivial steady state; without loss of generality, we omit independent variables in this discussion (i.e., *m* = 0 in Eq. 2), because they may be subsumed in the corresponding rate constants. The *k*^th^ equation is set to 0 and the terms associated with *X*_*k*_ are collected, which yields

4 $${\alpha }_{k}{X}_{k}^{{g}_{kk}-{h}_{kk}}={\beta }_{k}{\prod }_{j=1,j\ne k}^{n}{X}_{j}^{{h}_{kj}-{g}_{kj}}$$

and thus

5 $${X}_{k}={\left(\frac{{\beta }_{k}}{{\alpha }_{k}}{\prod }_{j=1,j\ne k}^{n}{X}_{j}^{{h}_{kj}-{g}_{kj}}\right)}^{1/({g}_{kk}-{h}_{kk})} .$$

This expression for *X*_*k*_ is substituted throughout the ODEs of the S-system in a *symbolic* manner. In other words, combinations of parameter values are not numerically merged or simplified. This retention of symbolic parameters in reduced models permits simulations with altered parameter values, indicates relationships among parameters, and provides hints regarding questions of identifiability; an example is shown below.

The S-system reduction is feasible in general, except for rare situations. First, if *X*_*k*_ does not appear in *any* of the equations, its ODE is already decoupled from the system and the remaining equations can be investigated without it. Second, if *X*_*k*_ appears in one or more equations, but not its own, or if *g*_*kk*_ = *h*_*kk*_, one starts the reduction with other equations, until *X*_*k*_ appears in its own equation. If this does not happen, the *k*^th^ ODE cannot be reduced.

**Fig. 2:**
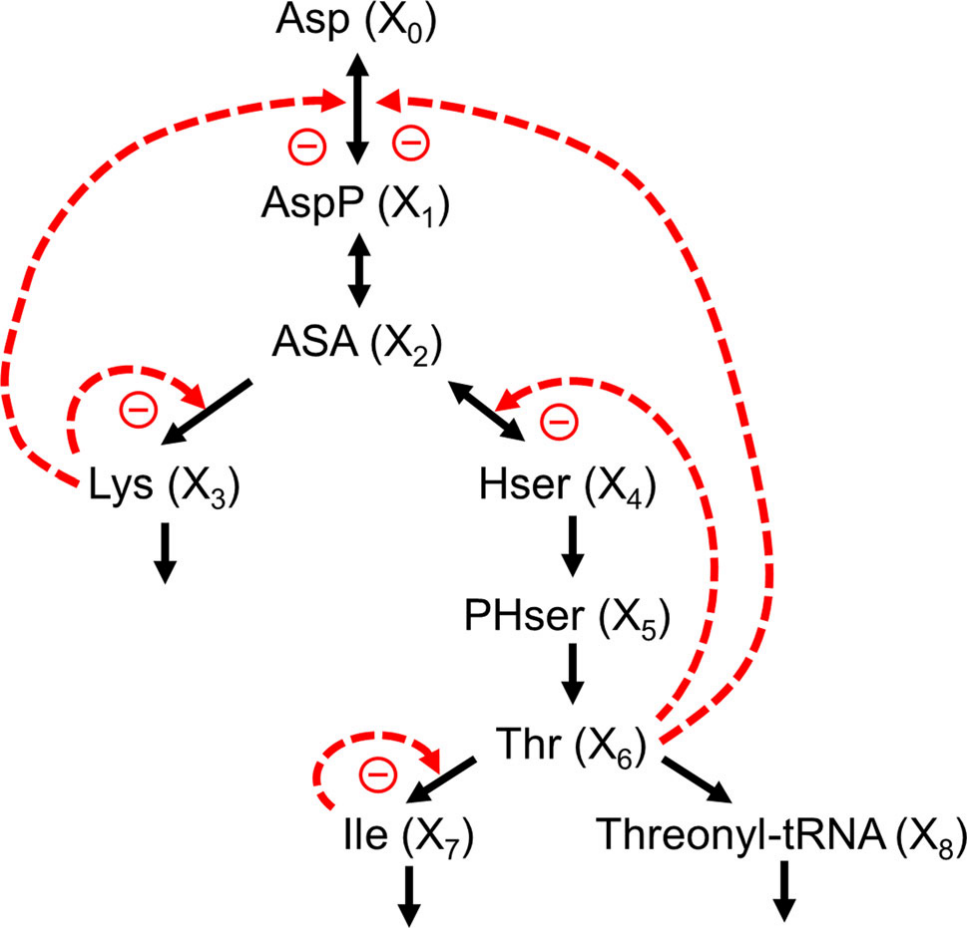
Metabolic reaction network of the biosynthesis of aspartate-derived amino acids (Curien et al. 2009). Abbreviations: Asp—L-Aspartate (independent variable *X*_0_), AspP—L-Aspartate-4-phosphate (*X*_1_), ASA—L-Aspartate-semialdehyde (*X*_2_), Lys—L-Lysine (*X*_3_), Hser—Homoserine (*X*_4_), PHser—O-Phospho-L-homoserine (*X*_5_), Thr—L-Threonine (*X*_6_), Ile—L-Isoleucine (*X*_7_), Threonyl-tRNA (*X*_8_).

As a detailed demonstration of the proposed model reduction strategy, consider the pathway of aspartate-derived amino acid synthesis (Curien et al. 2009) (Fig. 2). The model was originally formulated with a mixture of kinetic formats in the tradition of Michaelis and Menten, for which a direct systematic reduction would be difficult. However, Iwata et al. (2013) approximated the original model with an S-system and showed that the quality of this approximation was very good. The S-system model consists of eight ODEs, and the task is to reduce it by eliminating one or more equations. Details are presented in Supplement Section S1.

Curien’s paper (Curien et al. 2009) included inhibitory and activating effects of L-valine and S-adenosyl-methionine. However, these metabolites were considered constant in Iwata’s model and are therefore here subsumed in the corresponding rate constants. Iwata’s equations are as follows:

6 $$\begin{array}{ll} {\dot{X}}_{1} ={\alpha }_{1}{X}_{0}{X}_{3}^{{g}_{13}}{X}_{6}^{{g}_{16}}- {\beta }_{1}{X}_{1}^{{h}_{11}} & {\dot{X}}_{5} ={\alpha }_{5}{X}_{4}^{{g}_{54}}- {\beta }_{5}{X}_{5}^{{h}_{55}} \\ {\dot{X}}_{2} ={\alpha }_{2}{X}_{1}^{{g}_{21}}- {\beta }_{2}{X}_{2}^{{h}_{22}} & {\dot{X}}_{6} ={\alpha }_{6}{X}_{5}^{{g}_{65}}- {\beta }_{6}{X}_{6}^{{h}_{66}} \\ {\dot{X}}_{3} ={\alpha }_{3}{{X}_{2}^{{g}_{32}}X}_{3}^{{g}_{33}}- {\beta }_{3}{X}_{3}^{{h}_{33}} & {\dot{X}}_{7} ={\alpha }_{7}{{X}_{6}^{{g}_{76}}X}_{7}^{{g}_{77}}- {\beta }_{7}{X}_{7}^{{h}_{77}} \\ {\dot{X}}_{4} ={\alpha }_{4}{{X}_{2}^{{g}_{42}}X}_{6}^{{g}_{46}}- {\beta }_{4}{X}_{4}^{{h}_{44}} & {\dot{X}}_{8} ={\alpha }_{8}{X}_{6}^{{g}_{86}}- {\beta }_{8}{X}_{8}^{{h}_{88}} \\ \end{array}$$

Numerical specifications are provided in Supplement Section S1. Suppose that threonine (*X*_6_) is of particular interest and that variable *X*_3_, being on a separate branch, is not particularly important for the model dynamics, although its influence (feedback inhibition) should not be ignored. For the reduction, the equation for ${\dot{X}}_{3}$ is set equal to zero and *X*_3_ is expressed as a function of all other variables in this equation:

7 $${X}_{3}={\left(\frac{{\beta }_{3}}{{\alpha }_{3}}{X}_{2}^{-{g}_{32}}\right)}^{1/({g}_{33} - {h}_{33})}$$

*X*_3_ is subsequently substituted *symbolically* in this form for all instances of *X*_3_ in the remaining equations, thereby reducing the total number of equations by one.

Amazingly, this reduction process can be iterated with other variables until only the ODE of *X*_6_ is left; in the reduced form, it is called *Y*_6_. Variables *X*_1_–*X*_5_ are eliminated and *X*_7_ and *X*_8_ can be reduced to algebraic equations (now called *Y*_7_ and *Y*_8_). The result is

$$ \begin{gathered} \dot{Y}_{6} { } = \alpha_{6} ((\alpha_{5} ((\alpha_{4} ((\alpha_{2} (((\alpha_{1} Y_{0} ((\alpha_{3} ((\alpha_{2} (((\alpha_{1} Y_{0} ((\alpha_{3} ((\alpha_{2} (((\alpha_{1} Y_{0} ((\alpha_{3} ((\alpha_{2} \gg \hfill \\ \big( \big(\alpha_{1} Y_{0} \left( {\left( {\alpha_{3} \left( {\left( {\left( {\alpha_{2} / \, \beta_{2} } \right)^{(1/0.4)} / \, \beta_{3} } \right)} \right)^{(1/1.67)} } \right)^{(1/1.67)} } \right)^{ - 0.121} Y_{6}^{ - 0.163} / \gg \hfill \\ \end{gathered} $$

$$ \beta_{1} )^{(1/ \, 0.56106261)} )^{0.398} / \, \beta_{2} )^{(1/0.4)} )^{0.847} / \, \beta_{3} )^{(1/1.67)} )^{ - 0.121} Y_{6}^{ - 0.163} )/ \, \beta_{1} )^{2} )^{0.398} / \gg $$

$$ \begin{gathered} \beta_{2} )^{(1/0.4)} )^{0.847} / \, \beta_{3} )^{(1/1.67)} )^{ - 0.121} Y_{6}^{ - 0.163} )/ \, \beta_{1} )^{2} )^{0.398} / \, \gg \hfill \\ \beta_{2} )^{(1/0.4)} )^{0.847} / \, \beta_{3} )^{(1/1.67)} )^{ - 0.121} Y_{6}^{ - 0.163} )/ \, \beta_{1} )^{2} )^{0.398} / \, \gg \hfill \\ \end{gathered} $$

8a $$ \beta_{2} )^{(1/0.4)} )^{0.0982} Y_{6}^{ - 0.0251} / \, \beta_{4} )^{(1/0.1)} )^{0.092} / \, \beta_{5} )^{(1/0.1)} )^{1.02} - \, \beta_{6} Y_{6}^{0.1} $$

8b $$ Y_{7} = \left( {\alpha_{7} Y_{6}^{0.949} / \, \beta_{7} } \right)^{(1/2.49)} $$

8c $$ Y_{8} = \, \alpha_{8} Y_{6}^{ - 0.22} /\beta_{8} $$

Note that the symbols ≫ indicate that the term continues on the next line.

The only remaining ODE of the system represents threonine (*Y*_6_), whereas *Y*_7_ and *Y*_8_ are algebraic “add-ons” that do not affect *Y*_6_.

**Fig. 3:**
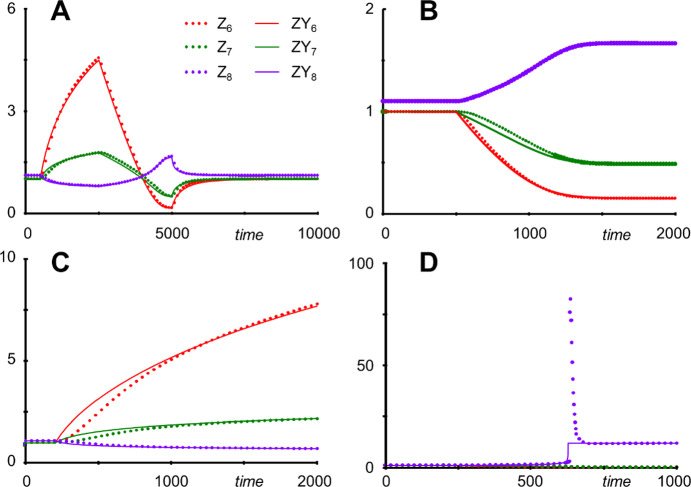
Comparison of Iwata's original S-system model (Iwata et al. 2013) of Currien's pathway system (Curien et al. 2009) with the reduced "system" in Eq. (8). For easier comparative display, all variables are normalized by their steady-state values; thus: *Z*_*i*_ = *X*_*i*_/*X*_*i_stst*_ and *ZY*_*i*_ = *Y*_*i*_/*Y*_*i_stst*_. **a** The simulation starts with an input of *X*_0_ = *Y*_0_ = 1. At times *t* = 500, 2500, and 5000, the input to the system is changed to 2, 0.5, and back to 1, respectively. **b** At *t* = 500, *b*_1_ is set from 5 to 10. **c** At *t* = 200, *a*_4_ is increased by 20%. **d** At *t* = 500, *b*_4_ is set from 5 to 10.

To assess the accuracy of the approximation, one may perform a typical simulation by changing the value of the input variable *X*_0_ (*Y*_0_) or of one or more of the parameters. The result is surprisingly good, even when the input is doubled or halved, which constitutes a significant perturbation (Fig. 3a). Similarly, changes in parameter values are generally mimicked rather well by the rduced system. For instance, a doubling of the degradation rate of *X*_1_, by setting *b*_1_ from 5 to 10 at *t* = 500, results only in a slight deviation in *Y*_6_, which is attenuated in *Y*_7_ and *Y*_8_, before the new steady state is reached (Fig. 3b). Indeed, most parameter changes have only mild effects on the systems dynamics (in both the original and the reduced forms). By contrast, increasing *a*_4_ or *a*_5_ even by 20% leads to much higher values in both the original and the reduced models (Fig. 3c). Nonetheless, both models approach the same steady state, which happens by design of the method. One notes that the dynamics of *Y*_6_ is in these cases slightly faster than in the original model. The biggest difference between *X*_6_ and *Y*_6_ results from doubling the degradation rate of *X*_4_, which is *b*_4_. Here, the reduced model misses a strong overshoot in *X*_8_ toward the new steady state, although both models again reach the same steady state (Fig. 3d).

Of course, one could algebraically convert the parameter combinations in the reduced Eq. (8) into numerical values. For the specific settings of the model (Supplements Section S1), one would obtain.

9 $$ \dot{Y}_{6} = {16}.{8}0{\mathrm{47Y}}_{0}^{{{1}.{634223}}} Y_{{6}}^{{ - 0.{5}0{19167}}} - 0.{5}Y_{{6}}^{{0.{1}}} . $$

One could furthermore subsume the external variable *Y*_0_ into the rate constant, which would yield the even simpler model.

10 $$\dot{Y}_{6} = {16}.{8}0{47}\;Y_{{6}}^{{ - 0.{5}0{19167}}} - 0.{5}Y_{{6}}^{{0.{1}}} .$$

Even though these formulations match the ODE in Eq. (8) exactly, this numerical simplification would no longer allow the modeling of targeted changes in parameter values or, in the latter case, the independent variable *Y*_0_, but only permit changes in the initial value of *Y*_6_.

One notes that the reduction of *X*_6_ does not yield results of a similar quality.

#### Reduction of GMA Models

GMA models do not guarantee algebraic nullcline computations. If they do, the reduction proceeds essentially as with S-system models. This will be demonstrated in the Sect. 4.

#### Reduction of LV Models

The size reduction of an LV model (Eq. 3) is straightforward and retains its canonical format. Suppose, for illustration, that *X*_*k*_ and the parameters *a*_*k*_ and *b*_*kk*_ in the *k*^th^ equation are not zero; details and other cases are discussed in Supplements Section S2. Setting the derivative ${\dot{X}}_{k}$ to 0 yields

11 $${-a}_{k}{X}_{k}=\left({\sum }_{j=1,j\ne k}^{n}{b}_{kj}{X}_{j}+ {b}_{kk}{X}_{k}\right){X}_{k}.$$

which can be solved for *X*_k_ upon division by *X*_k_. The result is:

12 $${X}_{k}={-a}_{k}{b}_{kk}^{-1}-{\sum }_{j=1,j\ne k}^{n}\frac{{b}_{kj}}{{b}_{kk}}{X}_{j}.$$

Substituting this expression in the remaining equations not only reduces the number of equations by 1 but also retains the LV format if the parameters are redefined:

13 $${\dot{X}}_{i}={\widetilde{a}}_{i}{X}_{i}+{\sum }_{j=1,j\ne k}^{n}{\widetilde{b}}_{ij}{{X}_{i}X}_{j}$$

with

14 $${\widetilde{a}}_{i}=\left({a}_{i }-{{a}_{k} b}_{ik}{ b}_{kk}^{-1} \right)$$

15 $${\widetilde{b}}_{ij}= {b}_{ij}- \frac{{{b}_{ik}b}_{kj}}{{b}_{kk}}$$

An example of LV-model reduction is shown in the Sect. 4.

## Results

The results will be documented with a series of representative examples that demonstrate the feasibility, successes and failures of canonical model reduction. As stated before, the original variables will always be called *X*_*k*_, while variables of the reduced system are denoted as *Y*_*k*_. This nomenclature permits easy comparisons and assessments of the model quality.

### Linear Pathway with Feedback

**Fig. 4:**
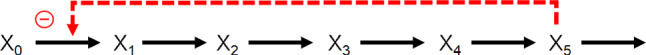
Five-variable linear pathway with external input and feedback inhibition.

As an introductory example, consider a five-variable linear pathway with external input and feedback inhibition by the end product (Fig. 4).

In S-system format, the model may be written as

$${\dot{X}}_{1} ={\alpha }_{1}{X}_{0}{X}_{5}^{g}- {\beta }_{1}{X}_{1}$$

$${\dot{X}}_{2} ={\beta }_{1}{X}_{1}- {\beta }_{2}{X}_{2}^{0.5}$$

16 $${\dot{X}}_{3} = {\beta }_{2}{X}_{2}^{0.5}- {\beta }_{3}{X}_{3}^{0.8}$$

$${\dot{X}}_{4} = {\beta }_{3}{X}_{3}^{0.8}- {\beta }_{4}{X}_{4}^{0.4}$$

$${\dot{X}}_{5} = {\beta }_{4}{X}_{4}^{0.4}- {\beta }_{5}{X}_{5}^{0.6}$$

The typical situation for linear metabolic pathways is that the intermediates have relatively low concentrations, because they serve no other purpose than being biochemically required for product formation. Furthermore, low-level intermediates allow enzymes to function with most of the flux directed forward and thus facilitate efficient flow of material and minimize metabolite load. They also prevent toxicity, unwanted side effects, and undue metabolic burden. Finally, low concentrations facilitate fine-tuned regulation of metabolic processes, because even modest changes in low intermediate concentrations can significantly affect the overall pathway flow. Based on these considerations, we choose the generic settings *X*_0_ = 1, *α*_1_ = 1, *β*_1_ = 0.2, *β*_2_ = 1.5, *β*_3_ = 3, *β*_4_ = 2, *β*_5_ = 0.4, and set the inhibition parameter as *g* =  − 1, indicating reasonably strong inhibition (see Chapter 5 of Voit (2000)). Note that a separation of time scales is not possible.

**Fig. 5:**
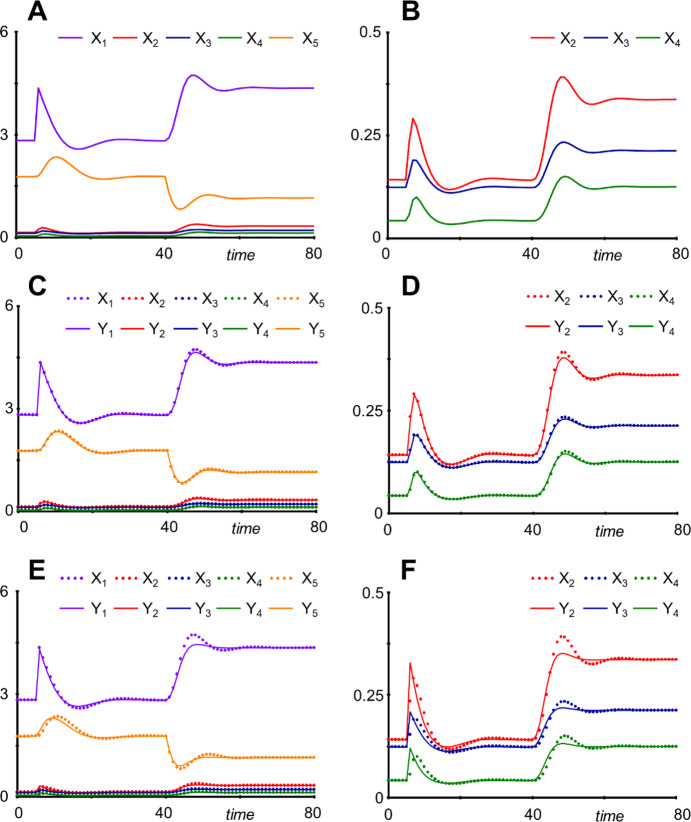
Simulations with the five-variable model of a linear pathway with external input and feedback inhibition. **a** The simulation starts at the steady state. Between *t* = 5 and *t* = 6, the input is increased from *X*_0_ = 1 to *X*_0_ = 4; furthermore, at time *t* = 40 the efflux from the system is doubled by setting *β*_5_ = 0.8. **b** The same simulations as in **a**, highlighting variables with smaller magnitudes. **c** and **d** The same simulation as in (**a**), but *X*_4_ was eliminated be reduction. **e** and **f** The same simulation as in (**a**), but *X*_2_, *X*_3_ and *X*_4_ were eliminated be reduction.

As an illustration of responses to perturbations, we start a simulation at the steady state (*X*_1*ss*_, …, *X*_5*ss*_) = (2.826188, 0.1419327, 0.1240745, 0.04240996, 1.766654), quadruple the input between *t* = 5 and *t* = 6 from *X*_0_ = 1 to *X*_0_ = 4, and then, at time *t* = 40, double the efflux from the system by setting *β*_5_ = 0.8. The change in efflux lowers the concentration of *X*_5_, which reduces the inhibition of the production of *X*_1_, leading to slight oscillations in the system variables and a rather different steady state (Fig. 5a, b).

Do demonstrate a reduction, we convert the fourth ODE from

17 $${\dot{X}}_{4} = {\beta }_{3}{X}_{3}^{0.8}- {\beta }_{4}{X}_{4}^{0.4}$$

to

18 $${Y}_{4} ={\left(\frac{{\beta }_{3}{Y}_{3}^{0.8}}{{\beta }_{4}}\right)}^{2.5}$$

Using the expression (18), *Y*_4_ may be eliminated entirely from the system, thereby reducing the number of variables and of parameter values that would have to be estimated from experimental data. In the present case, only the ODE for *Y*_5_ contains *Y*_4_. It thus becomes

19 $${\dot{Y}}_{5} = {\beta }_{4} {\left({\left(\frac{{\beta }_{3}{Y}_{3}^{0.8}}{{\beta }_{4}}\right)}^{2.5}\right)}^{0.4}- {\beta }_{5}{Y}_{5}^{0.6}={\beta }_{3}{Y}_{3}^{0.8}- {\beta }_{5}{Y}_{5}^{0.6} .$$

The ODEs for *Y*_1_, *Y*_2_, and *Y*_3_ correspond exactly to those in Eq. (18). The response of the reduced system is very similar to the original (Fig. 7c, d). In particular, the correct steady states are achieved, even if a parameter value is changed.

If all three intermediates are reduced, the expressions for *Y*_2_ − *Y*_4_ can be substituted sequentially into the ODEs (see Supplements Section S3), yielding the greatly reduced system

$${\dot{Y}}_{1} ={\alpha }_{1}{Y}_{0}{Y}_{5}^{g}- {\beta }_{1}{Y}_{1}$$

20 $${\dot{Y}}_{5} ={\beta }_{1}{Y}_{1}- {\beta }_{5}{Y}_{5}^{0.6}$$

The response of this reduced form is not dramatically different from the original (Fig. 7e, f), especially following the four-fold change in input between *t* = 5 and *t* = 6. In particular, the system, by design, always achieves the correct steady states, even if a parameter is permanently changed. Nonetheless, there is a noticeable different in the shape of the overshoots, following the doubling of efflux from the system at *t* = 40. This difference is the cost of the reduction, which yields a greatly simplifying approximation.

For this simple linear system, the equations resulting from the reduction (20) are not surprising: They simply indicate that the intermediates have been “ignored.”

## Bistable Systems

Padoan et al. (2021) state that the reduction of nonlinear models often leads to qualitatively different results. In particular, reduced models are not always able to capture multi-stability. To assess this situation for the proposed nullcline method, consider a bistable model of the form

$${\dot{X}}_{1} =4+8 \frac{{X}_{4}^{4}}{{4}^{4}+ {X}_{4}^{4}} {X}_{3}^{-0.5}- 0.5 {X}_{1}^{0.5}$$

$${\dot{X}}_{2} ={X}_{1}- 5 {X}_{2}^{0.5}$$

21 $${\dot{X}}_{3} =2 {X}_{2}- 3 {X}_{3}$$

$${\dot{X}}_{4} =3 {X}_{3}- 12 {X}_{4}^{0.75}$$

which was adapted from Voit (2005). It possesses three steady states (*X*_1*ss*_, …, *X*_4*ss*_), one of which is unstable:

1. 13.598, 7.3964, 4.9309, 1.3218 (stable)
2. 19.849, 15.760, 10.507, 3.6241 (unstable)
3. 27.251, 29.705, 19.804, 8.4381 (stable)

Note that the first equation is not in the format of a canonical model, but can be recast equivalently into this form (see Supplements Section S4). The reduction may be demonstrated with either version.

**Fig. 6:**
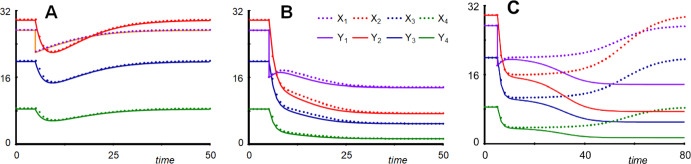
Simultaneous reduction of *X*_3_ and *X*_4_ in a bistable system. **a** The reduced system quite faithfully models the original if one starts the system at the high steady state and then lowers *X*_1_ to 22. **b** Similarly, the result is quite good for resetting *X*_1_ below the unstable point. **C** However, it is possible for the reduction to fail if *X*_1_ is set slightly above the unstable point, where the original returns to the high steady state but the reduced form decreases to the low steady state.

Starting the system at the upper steady state and resetting *X*_1_ to 22 at *t* = 5 shows a return to the same steady state, while resetting *X*_1_ to 16 causes the system to approach the low steady state. Simultaneous reduction of *X*_3_ and *X*_4_ to nullcline expressions for *Y*_3_ and *Y*_4_ essentially retains these results with modest deviations (Fig. 6a, b). Reduction of *X*_2_ also displays this behavior, but with a somewhat faster dynamics (not shown). Thus, the reduced model is able to capture bistability. However, the reduced system may not always be faithful: For instance, if *X*_1_ is reset to 18, close to the unstable steady state, the original model returns to the high steady state, due to *X*_2_, …, *X*_4_ being above the unstable point, while the reduced model approaches the low steady state (Fig. 6c). This discrepancy is caused by the approximate nature of the reduced model.

## Limit Cycles

**Fig. 7:**
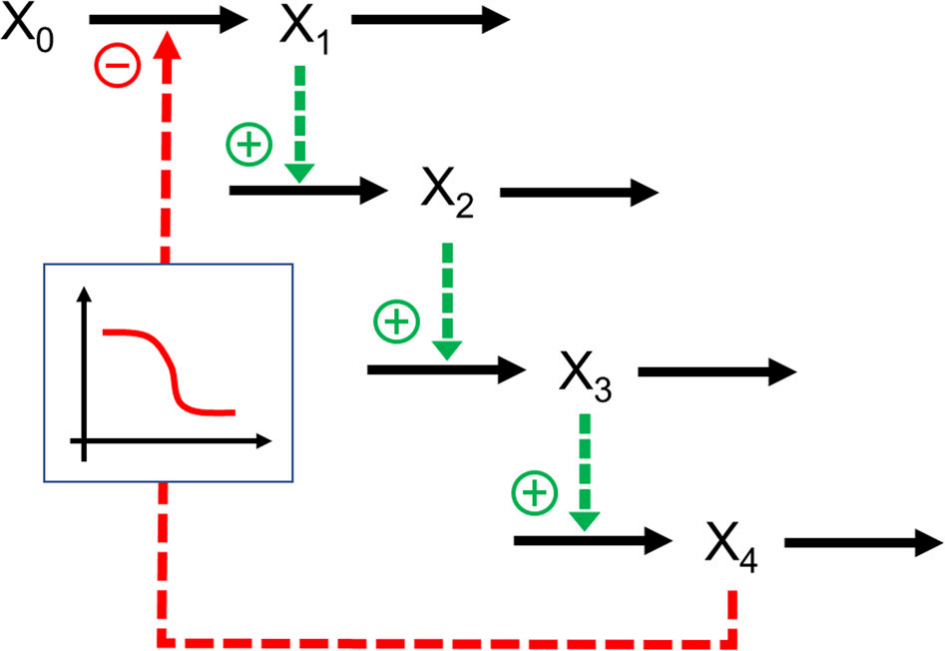
Goodwin's model (Gonze and Abou-Jaoude 2013; Goodwin 1965) of a cascade with feedback inhibition, here expanded by one additional layer.

Stable limit cycles typically oscillate around an unstable steady state. A model reduction therefore seems doubtful. Surprisingly, the reduction may retain the qualitative features of a limit cycle, but they can also differ substantially in numerical features. As an example, consider a classical cascading model with feedback, proposed by Goodwin over sixty years ago (Gonze and Abou-Jaoude 2013; Goodwin 1965) (Fig. 7). It is mostly a mass-action model and represents the inhibition with an inverse Hill function, ${k}_{1} \frac{{K}^{n}}{{K}^{n}+ {X}_{4}^{n}}$. The cascade is here expanded from three to four layers. The method of recasting converts this model into an equivalent S-system model (Supplements Section S5; (Savageau and Voit 1987)), by introducing the auxiliary variable ${X}_{5}= {X}_{4}^{n}+1$. The result is

22 $$ \begin{aligned}
  {\dot{X}}_{1} &=50 {X}_{5}^{-1} - 0.1 {X}_{1}\ \\ {\dot{X}}_{2} &=0.4{ X}_{1} - { X}_{2}\\ {\dot{X}}_{3} &={ X}_{2} - 0.1 { X}_{3} \\ {\dot{X}}_{4} &={ X}_{3} - 2 { X}_{4}\\ {\dot{X}}_{5} &=n{ X}_{3} {X}_{4}^{n-1} - 2 n {X}_{4}^{n} \end{aligned}$$

This recast S-system is used below to illustrate model reduction and to demonstrate that recasting does not impede this method. However, one could use the original model and obtain the same results.

**Fig. 8:**
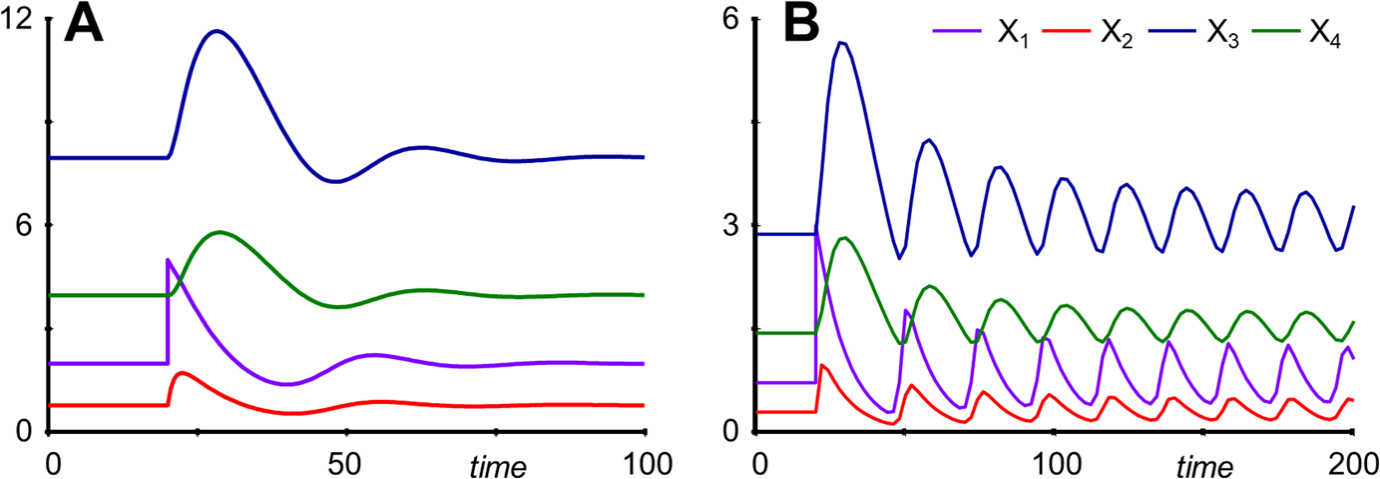
Depending on the value of the inhibition parameter *n*, Goodwin's model, as well as the 4-variable extension here, either displays damped oscillations (**a**) (*n* = 4) or stable limit cycle oscillations (**b**) (*n* = 18). In both cases, the system is started at its steady state and *X*_1_ is reset to 5 at *t* = 20.

As mentioned in Goodwin’s paper, the system exhibits damped oscillations toward a stable steady state ((*X*_1*ss*_, … *X*_4*ss*_) = (1.989, 0.7956, 7.956, 3.978)) for moderate values on *n* (e.g., *n* = 4) (Fig. 8a), whereas it enters stable limit cycle oscillations for large values of *n* (e.g., *n* = 18) (Fig. 8b), which surround an unstable steady state ((*X*_1*ss*_, … *X*_4*ss*_) = (0.71915, 0.28766, 2.8766, 1.4383)).

**Fig. 9:**
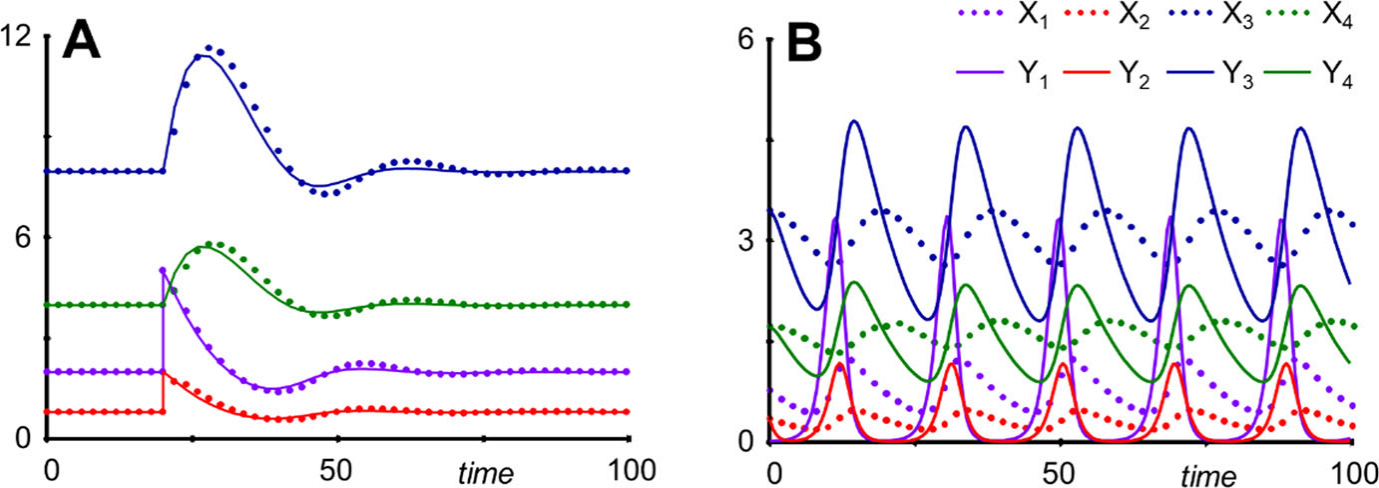
Simultaneous reduction of *X*_1_, *X*_4_ and *X*_5_ leads to a near-faithful reproduction of the original model's damped oscillations for *n* = 4 (**a**). **b** The same reduction for *n* = 18 retains the limit cycle behavior with the same frequency but very different amplitudes. In **a**, the system is started at its steady state and *X*_1_ is reset to 5 at *t* = 20. In **b**, the system does not have a stable steady state, and the system is started on the original system's limit cycle.

For *n* = 4, reductions of *X*_2_ or *X*_4_ and *X*_5_ generate very accurate results, and even reducing them simultaneously leads to an acceptable outcome, considering that *X*_1_ is reset from about 2 to 5 (Fig. 9a). By contrast, reducing *X*_1_ or *X*_3_ leads to substantial approximation errors, in the case of *X*_3_ essentially eliminating the oscillations (Supplements Section S5).

The situation for *n* = 18 is quite intriguing because reducing different variables has drastically different effects: If *X*_2_, *X*_3_, *X*_4_, or *X*_5_ are reduced, the limit cycle disappears, and the system displays damped oscillations (Supplements Section S5). However, if *X*_1_, or *X*_1_, *X*_4_, and *X*_5_ are reduced, the system generates a limit cycle with the correct frequency but with much larger amplitudes than the original (Fig. 9b).

## Chaotic Systems

Continuous-time chaotic systems require at least three first-order ODEs (Györgyi and Field 1992). Thus, reducing a three-variable system into a first-order system of lower dimension cannot retain chaotic features. In typical cases, such as the Lorenz system (Lorenz 1963), reduction attempts lead to unstable behavior or much simpler dynamics, such as rapidly damped oscillations toward a stable steady state.

**Fig. 10:**
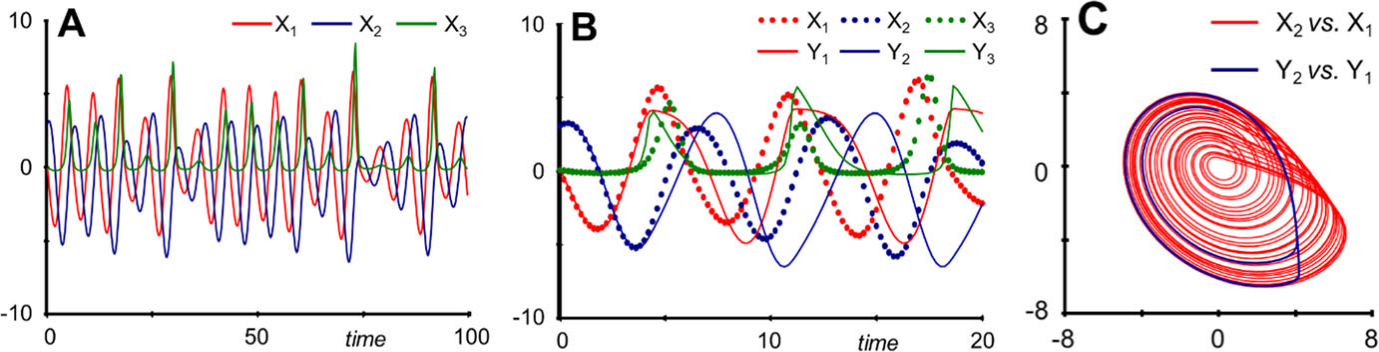
**a** Dynamics of Rössler's chaotic oscillator (23). **b** Upon reduction of *X*_3_, the system "tries" to mimic the chaotic trajectories, but instead approaches a limit cycle, which is best visualized in the phase plane (**c**). Interestingly, this limit cycle coincides with a projection of the boundary of the chaotic domain.

Intriguingly, reducing the third variable of the chaotic Rössler oscillator (Fig. 10a; (Rössler^1979^))

23 $$\begin{aligned} {\dot{X}}_{1} ={- X}_{2}- { X}_{3} & \quad { X}_{1}\left(0\right) =0 \\ {\dot{X}}_{2} ={ X}_{1}+0.36 { X}_{2} & \quad { X}_{2}\left(0\right) =3 \\  {\dot{X}}_{3} ={ 0.4 X}_{1}-4.5{ X}_{3}+ { X}_{1}{X}_{3} &  \quad { X}_{3}(0) =0  \end{aligned}$$

generates trajectories that initially mimic the chaotic trajectories (Fig. 10b), but then settles into a limit cycle that coincides with the projected boundary of the chaotic domain (Fig. 10c). Reducing the second variable does not show this behavior, but immediately starts to diverge (not shown).

Rössler proposed a more complicated four-variable system exhibiting “hyperchaos” (Rössler 1979):

24 $$ \begin{aligned} {\dot{X}}_{1} ={- X}_{2}- { X}_{4} & \quad { X}_{1}\left(0\right) =-20 \\  {\dot{X}}_{2} = { X}_{1} + 0.25 { X}_{2} + { X}_{3}
& \quad { X}_{2}\left(0\right) =0
\\ {\dot{X}}_{3} ={ 0.05 X}_{3}-0.5{ X}_{4} & \quad  { X}_{3}(0) =15 \\ {\dot{X}}_{4} =3+ { X}_{1}{ X}_{4} & \quad { X}_{4}\left(0\right) =0 \end{aligned}$$

Reduction of *X*_3_ again leads to a limit cycle, but with very large amplitude (not shown).

Harrington and Van Gorder (Harrington and Van Gorder 2017) reviewed reduction methods for chaotic systems and used differential elimination, based on sophisticated differential algebra, to reduce chaotic and hyperchaotic systems to lower-dimensional, but higher-order ODE. As an example, they transformed the Rössler system into a single, third-order ODE.

## Artificial Time-Scaling

**Fig. 11:**
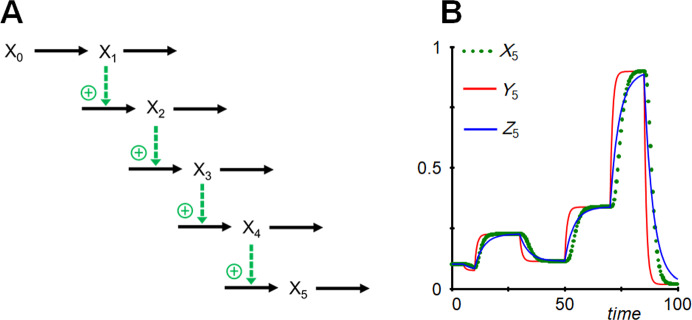
**a** Simple cascade in which each variable promotes the production of the subsequent variable. **b** Elimination of *X*_1_, …, *X*_4_ through reduction leads to a one-variable representation that faithfully attains the same new steady states as the original, if parameter values or the input are altered. However, it essentially eliminates time delays (*Y*_5_). Artificially slowing down time in the reduced system partially, but not fully, compensates for the speed-up (*Z*_5_).

By eliminating variables, especially those appearing in sequence, the dynamics of the reduced system tends to be faster than the original. This speed-up can to some degree be compensated with a scaling of time. As an example, consider the cascade in Fig. 11, where variables *X*_1_, …, *X*_4_ facilitate the generation of the subsequent variable; *X*_0_ is an independent input variable.

The cascade is straightforwardly represented with a mass-action model, which is a special case of an S-system:

25 $$ \begin{aligned} {\dot{X}}_{1}  ={ { a}_{1 }X}_{0}- { { b}_{1 }X}_{1} &\quad\quad { X}_{1}\left(0\right) =1\\ {\dot{X}}_{2} ={ { a}_{2 }X}_{1}- { { b}_{2 }X}_{2} & \quad\quad  { X}_{2}\left(0\right) =10\\ {\dot{X}}_{3} ={ { a}_{3 }X}_{2}- { { b}_{3 }X}_{3}  & \quad\quad { X}_{3}\left(0\right) =5 \\ {\dot{X}}_{4} ={ { a}_{4 }X}_{3}- { { b}_{4 }X}_{4}  &  \quad\quad { X}_{4}\left(0\right) =10\\ {\dot{X}}_{5} ={ { a}_{5 }X}_{4}- { { b}_{5 }X}_{5}  &\quad\quad { X}_{5}\left(0\right) =0.1  \end{aligned}$$

Reasonable, more or less arbitrary, parameter settings are *X*_0_ = 1, *a*_1_ = 1, *a*_2_ = 10, *a*_3_ = 0.5, *a*_4_ = 2, *a*_5_ = 0.01 and all *b* = 1. For a representative multi-step simulation, parameter values or the input variable are altered at different times, according to Table 1.

**Table 1:** Sequential changes in parameter values or input variables at different times

Time of change	5	10	30	50	70	85
Parameter change	*a*_1_ = 0.75	*a*_3_ = 1.5	*X*_0_ = *Y*_0_ = 0.5	*X*_0_ = *Y*_0_ = 1.5	*a*_1_ = 2	*a*_2_ = 2

Suppose now that variables *X*_1_, …, *X*_4_ have been eliminated through sequential reduction. The sole remaining equation of the reduced system is.

26 $${\dot{Y}}_{5} ={ { a}_{5 }({ a}_{4 }({ a}_{3 } ({ a}_{2 } ({ a}_{1 } Y}_{0}/{ b}_{1})/{ b}_{2})/{ b}_{3})/{ b}_{4})- { { b}_{5 }Y}_{5}.$$

The reduction, while attaining the correct new steady states, causes a noticeable speed-up in the simulated time course of *Y*_5_. This discrepancy can be remedied to some degree by artificially slowing down time; for comparison, the scaled variable is called *Z*_5_:

27 $${\dot{Z}}_{5} ={ { (a}_{5 }({ a}_{4 }({ a}_{3 } ({ a}_{2 } ({ a}_{1 } Z}_{0}/{ b}_{1})/{ b}_{2})/{ b}_{3})/{ b}_{4})- { { b}_{5 }Y}_{5})\cdot SF.$$

With *SF* = 0.25, the trajectory is much closer to the original dynamics (Fig. 11b).

## GMA-Systems: Metabolic Engineering using the White-Rot Fungus *Phanerochaete chrysosporium*

Hormiga et al. (2008) proposed a model for guiding the engineered production of biomass in the white-rot fungus *Phanerochaete chrysosporium*. This fungus is of great biological, environmental and biotechnological interest as it is capable of completely degrading the wood-forming polymer lignin, using specialized peroxidases. At the same time, it does not consume cellulose (Mycocosm 2025), which may subsequently be fermented into “second-generation” biofuels (Sims et al. 2010). Mathematical modeling is expected to play a role in society's push for such sustainable biofuels (Avinash and Murugesan 2018; Faraji et al. 2018; Silva et al. 2018).

The genome of *P. chrysosporium* indicates the presumed existence of over 100 cytochrome P450 monooxygenases (Martinez et al. 2004). These enzymes enable the fungus to degrade a wide variety of organic pollutants, including hazardous chemicals in soil and in water, such as phenol–formaldehyde (Gusse et al. 2006; Syed and Yadav 2012).

**Fig. 12:**
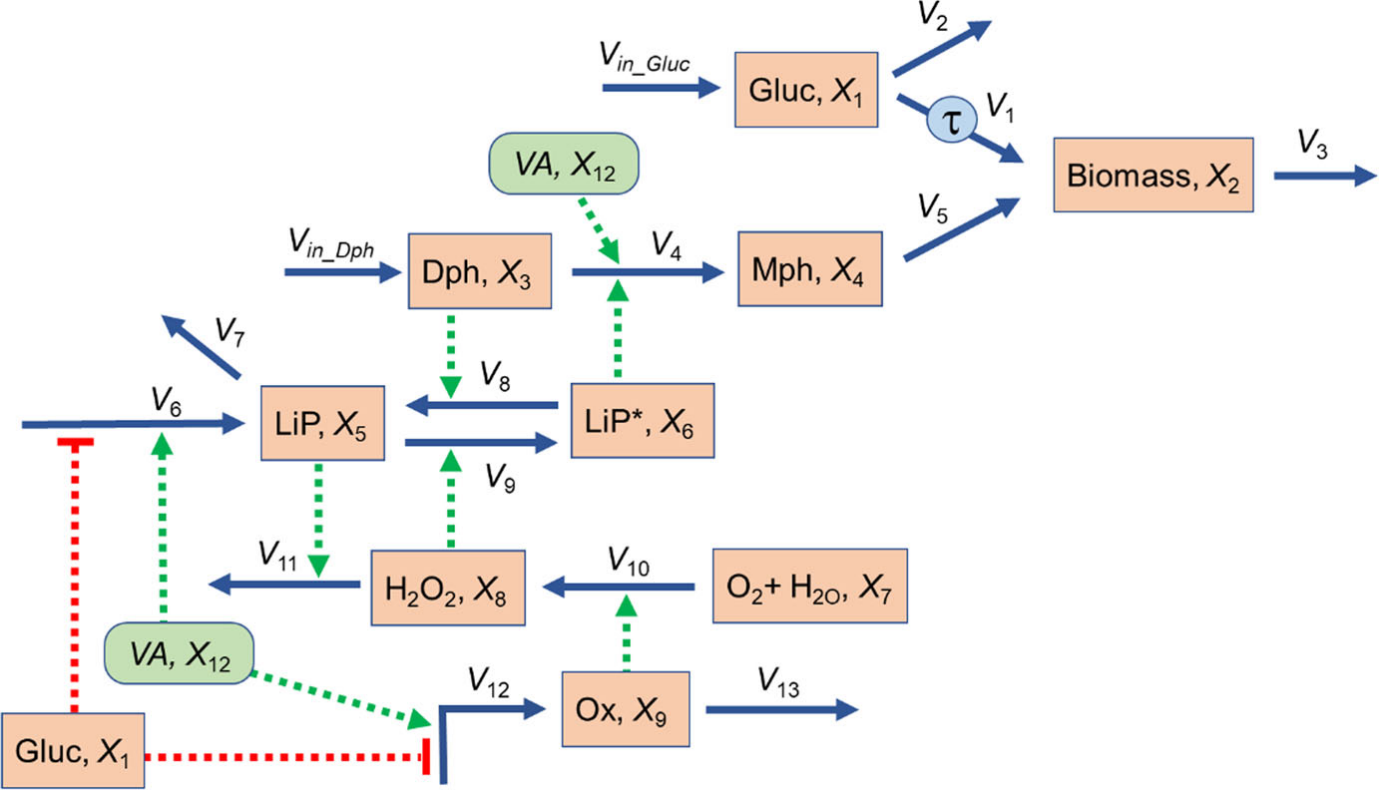
Diagram of biomass production in the white-rot fungus *Phanerochaete chrysosporium* as modeled by Hormiga et al. (2008). Abbreviations and variable names: Glucose (Gluc, *X*_1_); biomass (*X*_2_); diphenol (Dph, *X*_3_); monophenol (Mph, *X*_4_); lignin peroxidases in inactive form (LiP, *X*_5_); lignin peroxidases in activated form (LiP*, *X*_6_); O_2_ and H_2_O (*X*_7_); hydrogen peroxide (H_2_O_2_, *X*_8_); oxidase (Ox, *X*_9_); veratryl alcohol (VA, *X*_12_). *X*_10_ and *X*_11_ are not explicitly shown but used by Hormiga et al*.* as artificial variables causing the delay τ in flux *v*_1_.

Hormiga and collaborators geared their analysis toward understanding the regulatory control structure governing the increase in fungal biomass, with the ultimate goal of devising and optimizing a biotechnological set-up for the continuous operation of the ligninolytic system. Toward this end, they created a model representing the enzymatic sub-systems that drive biomass generation from two different substrates, as well as substrate consumption and the production of lignin peroxidase under a set of defined culture conditions. The scheme of this system is depicted in Fig. 12. It represents a biotechnological system where substrates are supplied externally. The model itself is presented in Supplements Section S6. Its base structure is that of a mass-action model, but accounting for regulation expands it to a GMA model.

Biomass (*X*_2_) is produced from two substrates, glucose (Gluc, *X*_1_) and monophenol (Mph, *X*_4_). The input substrates glucose and diphenol are supplied to the culture through fluxes *V*_*in_Gluc*_ and *V*_*in_Dph*_. The production from glucose is assumed to be delayed by τ, which the authors model with two auxiliary ODEs (*X*_10_, *X*_11_) (Macdonald 1978). Monophenol is generated from diphenol (Dph, *X*_3_) in a process that is catalyzed by activated lignin peroxidases (collectively called LiP*, *X*_6_) and enhanced by veratryl alcohol (VA, *X*_12_), which the authors consider to be an independent (externally provided) variable. LiP* is in balance with its inactive form (LiP, *X*_5_). The activation and deactivation processes are respectively affected by diphenyl and hydrogen peroxide (H_2_O_2_, *X*_8_) under the action of oxidase (Ox, *X*_9_) and degraded by lignin peroxidase (*V*_13_). H_2_O_2_ is generated from O_2_ and H_2_O, which are considered plentiful and modeled as a single independent (constant) variable (*X*_7_). The generation of both, lignin peroxidase and oxidase, is assumed to be affected by the amount of biomass, inhibited by glucose, and activated by veratryl alcohol. Further details may be found in Hormiga et al. (2008).

**Fig. 13:**
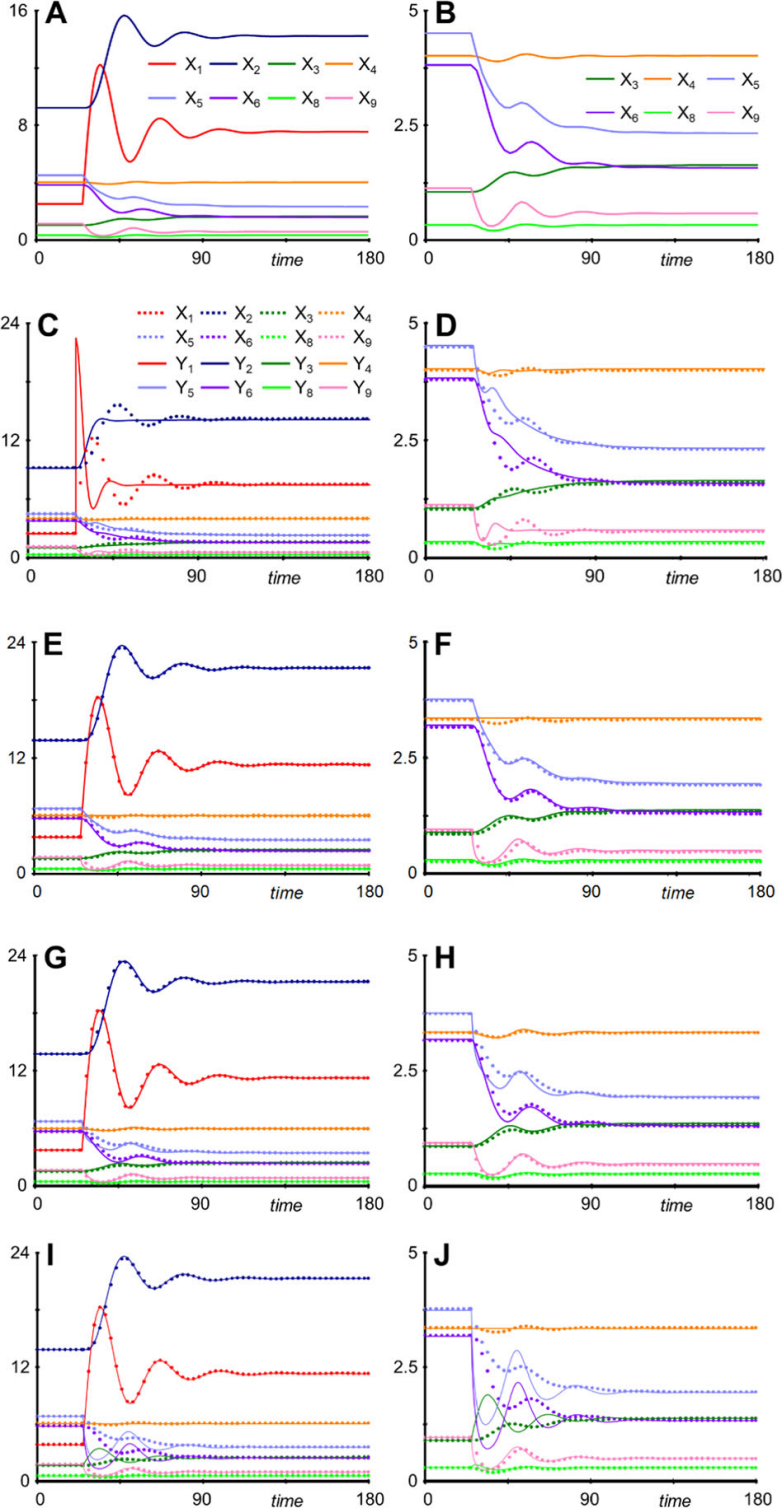
Responses of different variations of the white-rot fungus (WRF) model to a 200% increase in glucose input at *t* = 25. **a** Responses of all dependent variables of the base model. **b** Lower-magnitude metabolites of the base model. Note that *X*_7_ and *X*_12_ are considered constant (independent variables) and that *X*_10_ and *X*_11_ are artificial delay variables. **c** and **d** Consequences of reduction of *X*_1_ in the WRF model. The original oscillations are essentially lost. **c** All dependent variables. **d** Lower-magnitude metabolites. **e** and **f** Simultaneously reducing *X*_3_, *X*_4_, *X*_8_, and *X*_9_ in the WRF model has surprisingly little effect on the dynamics of the system. **e** All dependent variables. **f** Lower-magnitude metabolites. **g** and **h** Reducing *X*_5_ leaves glucose and biomass essentially unaffected, but causes noticeable perturbations in *Y*_3_ –*Y*_8_. **i** and **j** If only biomass is of interest, *X*_3_ –*X*_9_ can be reduced. The trajectories for *X*_1_ and *X*_2_ are almost exact; others not.

As a typical simulation, one may start the system at the steady state and reset the input of glucose, *V*_*in_Gluc*_, to 3, which corresponds to a 200% increase. In response to the perturbation, the system over- and undershoots before reaching a new steady state. Note that LIP (*X*_5_) and LIP* (*X*_6_), as well as Ox (*X*_9_) decrease due to the inhibition by glucose (Fig. 13a, b).

The task is now to test which variable reductions are feasible without undue consequences for the system dynamics; by design, the correct new steady-state values are always attained in these reductions. The first variables to examine are *X*_1_ (Gluc), *X*_10_ (artificial delay) and *X*_11_ (Gluc delayed). Reducing the first equation

28 $${\dot{X}}_{1} = { V}_{in\_Gluc }- { 0.3 X}_{11}- { 0.1 X}_{1}$$

to its nullcline yields

29 $${Y}_{1} =10 \left({ V}_{i{n}_{Gluc}}- { 0.3 Y}_{11}\right).$$

Replacement of the ODE for Y_1_ in the reduced white-rot fungus (WRF) model leads to a huge overshoot in the internal glucose concentration and to a dampening and alteration of oscillations (Fig. 13c, d). Reducing *X*_10_, *X*_11_, or both totally eliminates the oscillations caused by the time delay (Supplements Section S6).

Going through reductions of the other variables one by one (except for *X*_2_ (biomass), which is the variable of prime interest) reveals the following. Reducing *X*_3_ has almost no effect, except for a slight initial deviation in *Y*_4_. Similarly, reducing *X*_4_, *X*_8_, or *X*_9_ has essentially no visible effect on any of the trajectories (Supplements Section S6). Simultaneously reducing *X*_3_, *X*_4_, *X*_8_, and *X*_9_ also has very little effect; slight differences are seen in *Y*_4_ and *Y*_9_ (Fig. 13e, f). These results suggest that, not counting the delay variables and independent variable *X*_7_, the system can be reduced from *X*_1_–*X*_9_ to *X*_1_, *X*_2_, *X*_5_, and *X*_6_ without much effect on the dynamics. In other words, the number of required ODEs is roughly halved.

Reducing *X*_5_ leaves glucose and biomass essentially unaffected, but causes noticeable perturbations in *Y*_3_–*Y*_8_, highlighting the fact that the quality of the approximation by reduction depends on the variables of prime interest (Fig. 13g, h). Reducing *X*_6_ yields similar results to *X*_5_, although the differences are not quite as pronounced (Supplements Section S6).

Reducing *X*_3_ and *X*_4_ or *X*_5_ and *X*_6_ simultaneously slows down the solution, because the algebraic nullcline equations depend on each other. Solving these equations in terms of other variables remedies this issue of mutual dependence (Supplements Section S6).

If only biomass is of interest, *X*_3_–*X*_9_ can be reduced (Fig. 13i, j). The trajectories for *X*_1_ and *X*_2_ are still almost exact but others are clearly not. Reducing *X*_10_ and *X*_11_ as well eliminates overshoots and unduly speeds up the dynamics of all equations (Supplements Section S6).

### Lotka–Volterra Systems

Piccardi et al. (2019) studied competition among four bacterial species capable of degrading metal working fluids, which serve as substrates but are at the same time toxic. The species were *Agrobacterium tumefaciens* (*X*_1_), *Comamonas testosteroni* (*X*_2_), *Microbacterium saperdae* (*X*_3_) and *Ochrobactrum anthropi* (*X*_4_). Davis et al. (2022) used Piccardi’s bacterial growth data (especially the data in their supplementary figure S8) to develop and parameterize a four-variable Lotka–Volterra model of the type introduced in Eq. (5). Numerical details of this model are presented in Supplement Section S7.

In correspondence with Piccardi’s data, species *X*_2_ of Davis’ model is dominant for a while, but ultimately dies out. In other words, the model has no stable non-trivial steady state. A mere 20% increase in the growth rate of this species—*a*_1_ reset from 0.07440 to 0.08928—allows the system to reach such a non-trivial steady state. To illustrate LV-model reduction, this slightly altered model is used in the following. Comments regarding the original model can be found in Supplements Section S7.

**Fig. 14:**
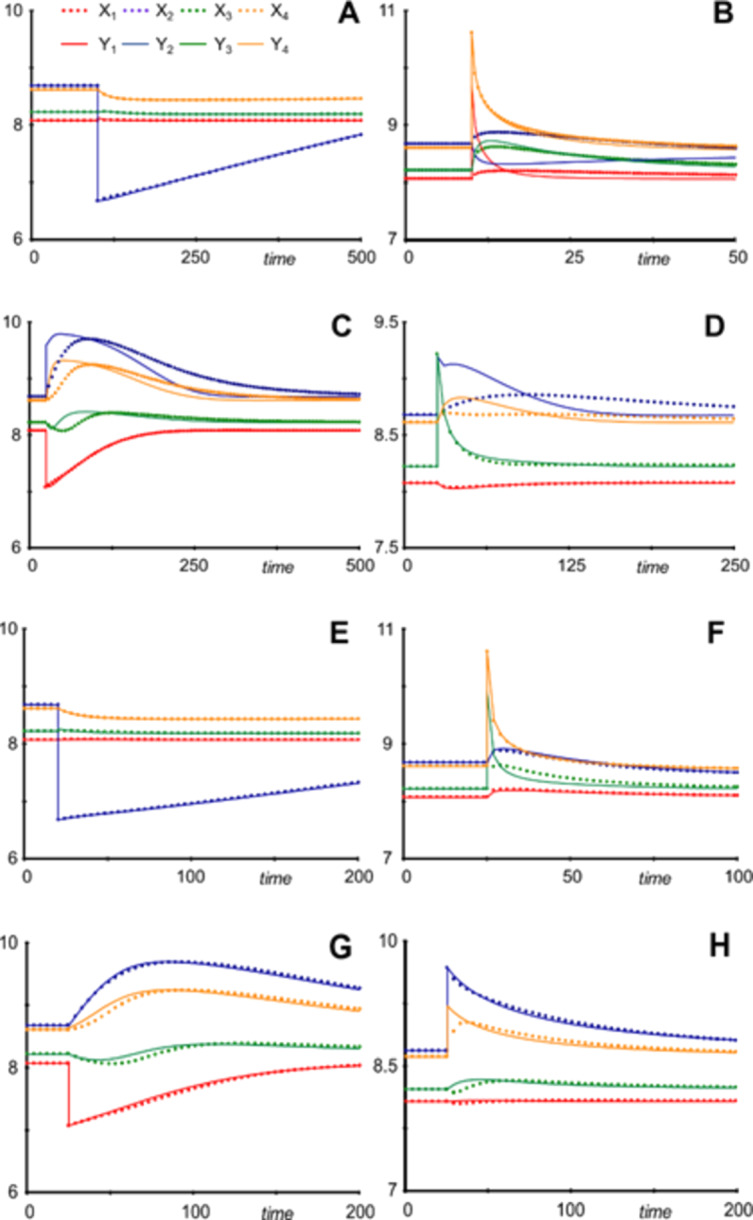
Responses to perturbations in differently reduced models of bacterial interactions (Table 2). **a** Reduction of *X*_1_ retains the original dynamics even after a 100-fold decrease in *X*_2_. **b** However, in response to a 100-fold increase in *X*_4_, the dynamics of *X*_2_ is "reversed": In the original, it slightly increases, whereas it decreases in the reduced form. The response in *X*_2_ is vastly stronger than in the original. **c** Reduction of *X*_2_ retains the original dynamics of *X*_1_ after a tenfold decrease in *X*_1_. The responses of the other variables are much faster than in the original. **d** Reduction of *X*_2_ recaptures the original dynamics of *X*_1_ and *X*_3_, but not *X*_2_ and *X*_4_, following a tenfold increase in *X*_3_. **e** Reduction of *X*_3_ yields essentially perfect results, when *X*_2_ is decreased 100-fold. **f** Reduction of *X*_3_ is similarly good for a 100-fold increase in *X*_4_. **g** Reduction of *X*_4_ yields acceptable results, here after a 100-fold decrease in *X*_2_. **h** Trajectories following a tenfold increase in *X*_2_ in a system with reduced *X*_2_.

Obviously, uncounted combinations of reductions and perturbations could be explored. Here, the results of a few strong, representative perturbations are shown. Employing the model reduction methods presented in the Sect. 3, the results are surprising in their variability (Fig. 14). In a nutshell: Reduction of *X*_1_ yields mixed results in trajectories that are sometimes excellent (perturbation in *X*_2_; Fig. 14a) but in other cases differ considerably from the original model trajectories (perturbation in *X*_4_; Fig. 14b). Reduction of *X*_2_ in many cases yields problematic results for substantial perturbations (Fig. 14c, d). By contrast, reducing *X*_3_ often yields essentially perfect trajectories, even under strong perturbations (Fig. 14e, f), and trajectories resulting from a reduction of *X*_4_ are often—but not always—acceptable. Simultaneously reducing *X*_3_ and *X*_4_ generally yields well-matched trajectories, similar to those reducing *X*_3_ or *X*_4_ individually (not shown). These results point to the dominant role of *X*_2_, while *X*_3_ and *X*_4_ are apparently less influential; *X*_1_ is somewhere in between.

**Table 2:** Simulation settings for Fig. 14

Panel	A	B	C	D	E	F	G	H
Reduced variable	*X*_1_	*X*_1_	*X*_2_	*X*_2_	*X*_3_	*X*_3_	*X*_4_	*X*_4_
Perturbation	*X*_2_ * 0.01	*X*_4_ * 100	*X*_1_ * 0.1	*X*_3_ * 10	*X*_2_ * 0.01	*X*_4_ * 100	*X*_1_ * 0.1	*X*_2_ * 10

## Toward a Theoretical Foundation

The proposed methods are effective but heuristic; they lack a solid theoretical foundation, beyond the fact that they are approximations that are accurate close to a chosen operating point, which here is the steady state of the system. It would clearly be very beneficial if rigorous a priori tests could reliably predict which reductions are or are not feasible within a predefined range of acceptability. So far, a “theory or feasible reducibility” has not emerged. Several avenues were pursued, leading to heuristic guidelines but not to rigorous criteria. For instance, it often occurs that variables or fluxes of low magnitudes can be reduced without a loss of accuracy. Then again, signaling systems are often designed to respond forcefully to low-magnitude triggers such as hormones binding to cell surface receptors.

One could also look at the magnitudes of the derivatives, which after all are set to zero. The issue here is that the slopes can change quite a bit, especially in the case of damped oscillations toward the steady state. Nevertheless, equating a derivative ${\dot{X}}_{k}$ with zero and solving for *X*_*k*_ in terms of the other variables corresponds to considering *X*_*k*_ as "temporarily constant," although it is not permanently constant, as it dynamically depends on the values of the other variables that are governed by ODEs. At each time point, the significance of this quasi-constancy could be analyzed with an *F*-test that would evaluate whether the variation in *X*_*k*_ is significantly less than that of other variables.

In several examples, a high relaxation speed toward the steady state indicated reasonable reducibility, but this criterion does not always hold. Similarly, dynamic sensitivities (Schwacke and Voit 2005; Shiraishi et al. 2005; Shiraishi and Hatoh 2007) were analyzed but did not lead to reliable predictions. Further research will be needed to disentangle the complexities of systems with respect to predictive reducibility.

To approach a true theoretical foundation, it might be possible to build upon the work of Tikhonov and Fenichel (Fenichel 1979; Klonowski 1983; Tikhonov 1952), which uses singular perturbation theory and permits a rigorous assessment of the quality of a model reduction. This work has been reviewed and explained in several later works (e.g., Eilertsen and Schnell 2020; Goeke and Walcher 2014; Goeke et al. 2015; Noethen and Walcher 2011; Verhulst 2005). Noethen and Walcher (2011) summarized the method in plain English: "Informally speaking, one hopes for the fast reactions or variables to run their course quickly, leaving a reduced system moving on some slow manifold." A conceptually similar idea was proposed by Savageau (1979).

The starting point of the Tikhonov-Fenichel approach is the distinction of variables according to their slow and fast dynamics, followed by the identification of a stable slow manifold, which is a lower-dimensional surface within the state space of the full system. The slow manifold represents the long-term behavior of the system, once the fast dynamics is essentially zero. The Tikhonov-Fenichel reduction (TFR) provides an algebraic method for determining the equations representing the slow manifold and for deriving the reduced model. The key is to let a time-scale parameter ε go to zero and solve the resulting fast nullcline equation. This solution is substituted into the slow equation, and the right-hand side of this equation is expanded into a Taylor series in ε (Eilertsen and Schnell 2020; Noethen and Walcher 2011). The first term of this expansion corresponds to the slow manifold. Higher-order terms of this expansion may be used to assess the quality of the approximation.

TFR is not directly applicable to the heuristic reduction proposed here, because the heuristic method does not involve time-scale separation. In fact, we analyzed examples where all variables operated at the same time scale. Nonetheless, one could conceptually consider each reduction nullcline as a quasi-steady state, for which much TFR work has been done (e.g., Eilertsen and Schnell 2020). This approach could possibly identify variables that are particularly well suited for reduction. An encouraging aspect is the critical assumption in TFR that the nullcline equation can be solved explicitly, which is certainly the case for S-system and LV models, as was demonstrated above.

## Variable Selection

A related but slightly different question within this context is the order of reductions, which could become an important issue if the reduction process is automated. On the one hand, the reduction process is commutative in a sense that the removal of a set of variables in any order will lead to the same reduced model. However, the situation is different in an exploratory study without a clear understanding of which variables should be removed or retained. In this case, the best order of removing equations, and when to stop, appear to be questions that should be addressed.

As an example, assume that a fully specified S-system model is available and that a certain dynamic behavior is of interest, e.g., in response to a particular perturbation. It is straightforward to write down multiple versions of reduced models, where each has a different variable—or set of variables—removed. One could then evaluate the quality of the reduced models in comparison with the original model and rank-order them by how well they match the original model's behavior. Turning the argument around, the elimination of different sets of variables would indicate the importance of these sets for the dynamics of the original model, at least with respect to specific perturbations. Ultimately, optimally chosen reductions would aid the effort of fine-tuned parameter estimation, if suitable data were available, as well as computational costs, especially for analyses of large-scale models.

A more systematic approach could be an extension of state sensitivity analysis, as proposed by Perumal et al. (2009). The method is based on Green's function matrix, which can be used to solve differential equations and assess parameter sensitivities (Arfken and Wber 2005; Varma et al. 1999). Specifically, the matrix contains sensitivity coefficients characterizing changes in dependent variables in response to perturbations at different initial times (Turányi 1990). The method could potentially be used to compute how strongly given trajectories depend on other state variables throughout a simulation. Variables consistently associated with low sensitivities could be considered prime candidates for reduction, thereby establishing a ranking for iterative reduction.

## Discussion

Throughout the foreseeable future, systems biologists will enjoy novel opportunities and face growing challenges. Many of these will be associated with the increasing sizes of models, which reflect the growing amounts of data and information becoming available through modern experimentation, including the various -omics techniques and the search for medical digital twins (Tudor et al. 2025). In addition to increased technical requirements in biomedical modeling, taxing issues will concern the relevance of specific data. Over the past twenty years, the biomedical community at large has been encountering an issue not seen before: unprecedented volumes of data became available but not all of them were of true value (Voit 2002). In the context of modeling, good data selection is crucial because every new variable and every new process in a model is associated with new parameters that require the specification of values for further analyses and simulations. While artificial intelligence is expected to aid the model design process in various ways, conceptual strategies must be developed to tame these demands of growing models.

One such strategy might utilize a combination of expert opinion and default parameter values. Subject area experts very often have a keen intuition for the subject of their investigation, but this type of semi-qualitative insight has largely been underutilized—if not entirely ignored—by the modeling community. The reason might be that “intuition” does not sound very scientific. Nonetheless, we used this strategy successfully to set up the first models of dopamine metabolism in the human brain, where specific metabolite concentrations and flux split ratios had not been documented in the literature (Qi et al. 2008a, b, 2010). Specifically, we asked experts regarding the concentrations of minor metabolites (e.g., dopamine quinone) relative to those of better characterized concentrations, such as L-DOPA. Similarly, we asked whether the fluxes diverging at branch points were of similar or rather different magnitudes. Answers like “Flux B is maybe 10% of Flux A, but it could be 8% or 15%, we just don’t know” were initially taken as facts. Complementing this information, we used power-law models with default parameter values (e.g., kinetic orders of 0.5 and − 1 for substrates and inhibitors, respectively; Chapter 5 in Voit (2000)). With some finetuning, the models became surprisingly accurate, at least in a semi-quantitative manner (e.g., Qi et al. 2016). One reason for this surprising quality, purely based on heuristics, is that high precision in parameter values is actually seldom needed, as long as the model structure is correct. Beyond these types of questions, one may even retrieve parameter information from asking experts what time trends they would expect in a given variable, following a stimulus (Goel et al. 2006).

Once a coarse initial model is constructed, it would be beneficial to reduce its size, for instance, with methods introduced here. Such a reduction simplifies the analysis in various ways. Not even mentioning lowered technical needs and issues like stiffness in large systems (Hairer and Wanner 1996), the reduction analysis might point to those variables that are true drivers of the model dynamics and those that only play peripheral roles. Crystallizing these variables could also be a first step toward the discovery of motifs, design and operating principles in biomedical systems (Alon and An 2019; Alves and Savageau 2000c; Irvine and Savageau 1985; Lee et al. 2011; Milo et al. 2002; Savageau 1985, 1989; Voit 2004). The reduction would also facilitate parameter estimation, which is notoriously hampered by insufficient data, especially for variables of secondary interest, such as metabolic intermediates.

If canonical models like S-systems and LV-models are employed, the reduction analysis can be simplified with relative ease because every reduction step is formulaic, as long as some basic requirements are satisfied (see Sect. 3). Thus, specifying which variables are of prime interest and should be retained and which typical perturbations needed to be addressed, an algorithm could systematically check which other variables could be eliminated without undue loss of output quality. Indeed, alternative reductions of the same model would demonstrate how strongly the dynamics of a system is affected by any combinations of its variables. This type of automation raises questions of reduction feasibility and the order of reductions, as discussed before.

Prominent types of models that will greatly benefit from reduction are hybrid and Agent-Based Models (ABMs) in which some of the agents are governed by ODE systems (e.g., Bodine et al. 2020; Cilfone et al. 2015; Cruz and Kemp 2022; Fonseca et al. 2025; Glen et al. 2019). Due to the nature of ABMs, the ODEs must be solved thousands of times, sometimes with slightly varied parameter values, in which case the method proposed here compares favorably with setting variables constant.

Even within the realm of pure ODE models, repeated solutions have become a standard tool, for instance, in Monte-Carlo simulations, where the same model is to be solved many thousands of times with slightly varied parameter values (Harrison 2010; Velikova et al. 2024). These tools are particularly important for simulations of complex multi-scale and ensemble models that defy explicit analyses. Within this context, it is quite evident that a high-level model should not necessarily have to carry all sub-models along all the time and in complete detail, especially under “normal” physiological conditions (Kumbale et al. 2025, 2021). Instead, it would be very useful to minimize sub-models to dynamic models that are simplified as much as possible, without losing critical information. Such a valid simplification becomes obligatory for diagnosis, explanation and prediction, if models acquire large sizes.

The heuristic reduction methods proposed here are straightforward. Nonetheless, much remains to be done to make them mathematically rigorous. One prong of future analysis could be the search for crisper a priori criteria of valid reducibility. A second prong could be the creation of effective algorithms for automatic, iterated reduction. Most important, however, will be the establishment of a theoretical foundation on which the heuristic methods rest. A starting point could be the work of Tikhonov and Fenichel (Eilertsen and Schnell 2020; Fenichel 1979; Goeke and Walcher 2014; Goeke et al. 2015; Klonowski 1983; Noethen and Walcher 2011; Tikhonov 1952; Verhulst 2005). To pursue this avenue, one would have to define a time-scale parameter ε that might consist of a combination of system parameters and would approach zero when the system approaches its slow manifold. This approach would ideally be combined with a systematic ranking of variables for reduction, thereby yielding a "reduction pipeline," based on sensitivity analysis that characterizes the effects of impulse perturbations on the dependent variables (Perumal et al. 2009).

## Supplementary Information

Below is the link to the electronic supplementary material.

Supplementary file1 (DOCX 986 KB)

## Data Availability

The article only uses secondary data from published articles.
