# Supplementary material for: Format-Preserving Reduction of Canonical Nonlinear Models
Source: Bull Math Biol. 2026 Mar 4;88(4):49. doi: 10.1007/s11538-026-01599-2 (PMC12960407; doi:10.1007/s11538-026-01599-2)
Supplement: Supplementary file 1 — Supplementary file1 (DOCX 986 KB) [file 11538_2026_1599_MOESM1_ESM.docx]

**Supplements:**

**Format-Preserving Reduction of Canonical Nonlinear Models**

**Eberhard O. Voit**

The supplements contain parameter values and other information regarding the models presented in the *Main Text*, as well as additional figures. As in the *Main Text*, variables of the original models are denoted with indexed *X* variables, while the corresponding reduced variables are designated with indexed *Y*s.

**Supplement Section S1: Model of Aspartate-Derived Amino Acid Synthesis**

The metabolic reaction network of aspartate-derived amino acid synthesis [1] is shown in the *Main Text* (Figure 2). The original model was formulated with traditional rate functions, but Iwata and colleagues [2] converted it into an S-system and showed that the quality of this approximation was very good. They also added as explicit variables *X*_7_ = [Ile] and *X*_8_ = [Threonyl-tRNA]. In the original model, *X*_8_ had no explicit efflux and therefore continued to accumulate without a steady state. Here, a default efflux term was added. The model structure is given in the *Main Text*. The parameter values are presented in Table S1; the value of the input variable, aspartate (*X*_0_), was set to 1. The initial values correspond to the non-trivial steady state of the system.

**Table S1.** Parameter settings for the model of aspartate-derived amino acid synthesis

|  | ***X*_1_** | ***X*_2_** | ***X*_3_** | ***X*_4_** | ***X*_5_** | ***X*_6_** | ***X*_7_** | ***X*_8_** |
| --- | --- | --- | --- | --- | --- | --- | --- | --- |
| ***Steady-State***  ***Value*** | 0.3384 | 0.9691 | 70.05 | 0.9216 | 44.91 | 343.7 | 62.69 | 0.254 |
| ***α*** | 12.6 | 15.2 | 124 | 5.76 | 7.37 | 0.0185 | 11.7 | 0.0919 |
| ***β*** | 5 | 10 | 0.1 | 5 | 5 | 0.5 | 0.1 | 0.1 |
| ***g*** | *g*_13_ = -0.121  *g*_16_ = -0.163 | *g*_21_ = 0.398 | *g*_32_ = 0.847  *g*_33_ = -1.37 | *g*_42_ = 0.0982  *g*_46_ = -0.0251 | *g*_54_ = 0.092 | *g_6_*_5_ = 1.02 | *g*_76_ = 0.949  *g*_77_ = -2.19 | *g*_86_ = -0.22 |
| ***h*** | *h*_11_ = 0.5 | *h*_22_ = 0.4 | *h*_33_ = 0.3 | *h*_44_ = 0.1 | *h*_55_ = 0.1 | *h*_66_ = 0.1 | *h*_77_ = 0.3 | *h*_88_ = 1 |

**Supplement Section S2:** **Reduction of Lotka-Volterra Models**

A Lotka-Volterra (LV) System with real-valued parameters can be written generically as

$$\dot{X}_{i}=a_{i}X_{i}+\sum_{j=1}^{n} b_{ij}{X_{i}X}_{j} (S1)$$

The task at hand is reduction of variable *X_k_*, assuming its nullcline equation $\dot{X}_{k}=0$ has a non-zero solution for *X_k_*. Depending on *a_k_* and *b_kk_*, the reduction proceeds slightly differently or cannot be performed.

***Case 1:* *a_k_* ≠ 0, *b_kk_* ≠ 0**

This case is discussed in *Main Text*, but details of the reduction follow below. Separating out the linear term of the *k*^th^ equation for $\dot{X}_{k}=0$ yields

$${-a}_{k}X_{k}=\left( \sum_{j=1,j\neq k}^{n} b_{kj}X_{j}+ b_{kk}X_{k} \right)X_{k}. (S2)$$

Division by *X_k_* simplifies this term to

$$-a_{k}=\left( \sum_{j=1,j\neq k}^{n} b_{kj}X_{j}+ b_{kk}X_{k} \right) (S3)$$

and

$$-b_{kk}X_{k}=a_{k} + \sum_{j=1,j\neq k}^{n} b_{kj}X_{j}, (S4)$$

which yields an algebraic term for *X_k_*:

$$X_{k}={-a}_{k}b_{kk}^{-1}-\sum_{j=1,j\neq k}^{n} \frac{b_{kj}}{b_{kk}}X_{j} (S5)$$

*X_k_* is a linear function of the other X-variables. As an example, substitute it in the equation for *X_i_*:

$$\dot{X}_{i}=a_{i}X_{i}+\sum_{j=1,j\neq k}^{n} b_{ij}{X_{i}X}_{j}+ b_{ik}X_{i} X_{k}. (S6)$$

Substitution of *X_k_* yields

$$\dot{X}_{i}=a_{i}X_{i}+\sum_{j=1,j\neq k}^{n} b_{ij}{X_{i}X}_{j}+ b_{ik}X_{i} \left( {-a}_{k}b_{kk}^{-1}-\sum_{j=1,j\neq k}^{n} \frac{b_{kj}}{b_{kk}}X_{j} \right), (S7)$$

$$\dot{X}_{i}=\left( a_{i}-{a_{k} b}_{ik}b_{kk}^{-1} \right)X_{i}+\sum_{j=1,j\neq k}^{n} b_{ij}{X_{i}X}_{j}- b_{ik}X_{i} \left( \sum_{j=1,j\neq k}^{n} \frac{b_{kj}}{b_{kk}}X_{j} \right), (S8)$$

$$\dot{X}_{i}=\left( a_{i}-{a_{k} b}_{ik}b_{kk}^{-1} \right)X_{i}+\sum_{j=1,j\neq k}^{n} b_{ij}{X_{i}X}_{j}- \left( \sum_{j=1,j\neq k}^{n} \frac{{b_{ik}b}_{kj}}{b_{kk}}{X_{i}X}_{j} \right), (S9)$$

and finally

$$\dot{X}_{i}=\left( a_{i}-{a_{k} b}_{ik}b_{kk}^{-1} \right)X_{i}+\sum_{j=1,j\neq k}^{n} {(b}_{ij}- \frac{{b_{ik}b}_{kj}}{b_{kk}}){X_{i}X}_{j}, (S10)$$

which is in the LV format

$$\dot{X}_{i}=\tilde{a}_{i}X_{i}+\sum_{j=1,j\neq k}^{n} \tilde{b}_{ij}{X_{i}X}_{j} (S11)$$

with

$$\tilde{a}_{i}=\left( a_{i}-{a_{k} b}_{ik}b_{kk}^{-1} \right) (S12)$$

and

$$\tilde{b}_{ij}= b_{ij}- \frac{{b_{ik}b}_{kj}}{b_{kk}} . (S13)$$

***Case 2:* *a_k_* = 0, *b_kk_* ≠ 0**

For *X_k_* ≠ 0, the nullcline equation $\dot{X}_{k}=0,$

$$0=\sum_{j=1}^{n} b_{kj}{X_{k}X}_{j}, (S14)$$

may be divided by *X_k_*, which results in

$$0=\sum_{j=1}^{n} b_{kj}X_{j}. (S15)$$

Separating the term containing *X_k_* yields

$$0=\sum_{j=1,j\neq k}^{n} b_{kj}X_{j}+ b_{kk} X_{k}, (S16)$$

and thus

$$X_{k}= -\sum_{j=1,j\neq k}^{n} \frac{b_{kj}}{b_{kk}}X_{j}, (S17)$$

which is substituted into all other ODEs, thereby retaining the LV format.

***Case 3:* *a_k_* = 0, *b_kk_* = 0**

For *X_k_* ≠ 0, the nullcline equation

$$\dot{X}_{k}=a_{k}X_{k}+\sum_{j=1}^{n} b_{kj}{X_{k}X}_{j} =0 (S18)$$

reduces to

$$0=\sum_{j=1}^{n} b_{kj}{X_{k}X}_{j}, (S19)$$

$$0=\sum_{j=1}^{n} b_{kj}X_{j}, (S20)$$

and thus

$$0=\sum_{j=1,j\neq k}^{n} b_{kj}X_{j}. (S21)$$

This equation cannot be solved for *X_k_*. Expressed in words, if the ODE for *X_k_* does not contain *X_k_* itself, it cannot be reduced.

**Supplements Section S3: Linear Pathway with Feedback**

Successive reduction of the linear system with feedback ultimately leads to a system of only two equations. The *Main Text* demonstrated reduction of the 4^th^ variable and substitution into the 5^th^ ODE, yielding

$\dot{Y}_{5} = \beta_{4} {({(\frac{\beta_{3}Y_{3}^{0.8}}{\beta_{4}})}^{2.5})}^{0.4}- \beta_{5}Y_{5}^{0.6}=\beta_{3}Y_{3}^{0.8}- \beta_{5}Y_{5}^{0.6}$. (S22)

Substituting *Y*_3_ gives the nullcline equation

$\dot{X}_{3} = \beta_{2}X_{2}^{0.5}- \beta_{3}X_{3}^{0.8}=0$ (S23)

which is solved as

$Y_{3} ={(\frac{\beta_{2}Y_{2}^{0.5}}{\beta_{3}})}^{1/0.8}$ (S24)

Substituting this expression in the equation for *Y*_5_ leads to

$\dot{Y}_{5} =\beta_{2}Y_{2}^{0.5}- \beta_{5}Y_{5}^{0.6}$ (S25)

Similarly, reducing *X*_2_ and substituting *Y*_2_ yields

$\dot{Y}_{5} =\beta_{1}Y_{1}- \beta_{5}Y_{5}^{0.6}$ (S26)

Thus, the reduced system is

$\dot{Y}_{1} =\alpha_{1}Y_{1}Y_{5}^{g}- \beta_{1}Y_{1}$ (S27a)

$\dot{Y}_{5} =\beta_{1}Y_{1}- \beta_{5}Y_{5}^{0.6}$ (S27b)

It indicates that, in this case, the low-level intermediates are simply "ignored."

**Supplements Section S4: Bistable Systems**

The first equation of the bistable system in the *Main Text* contains an expression in Hill-function format [3]. To convert it equivalently into a power-law equation, using the recasting technique [4], one defines the auxiliary variable

$X_{5}= 4^{4}+ X_{4}^{4}$. (S28)

Its derivative is

$$\dot{X}_{5} = 4 X_{4}^{3} \dot{X}_{4} =4 X_{4}^{3} \left( 3 X_{3} - 12 X_{4}^{0.75} \right),$$

$X_{5}\left( 0 \right)=4^{4}+ X_{4}^{4}\left( 0 \right)$ (S29)

which is in power-law format. Substituting *X*_5_ (S28) into the equation of *X*_1_ yields a GMA system.

This system could be recast further into an S-system, for instance, by replacing *X*_1_ with the ratio of two auxiliary variables: *X*_1_ = *X*_6_/*X*_7_ [4, 5]. *X*_1_ is thereby eliminated from the system, leading to six S-system equations (*X*_2_, …, *X*_7_) instead of four in the original.

An interesting alternative is to *approximate* $F= 4+8 \frac{X_{4}^{4}}{4^{4}+ X_{4}^{4}}$ with a power-law term of the type ${\alpha X}_{4}^{g}$. According to the principles of the power-law approximation [6], the kinetic order *g* is given as $g= \frac{dF}{dX_{4}}\frac{X_{4}}{F}$ and *α* as $F X_{4}^{-g};$ both are to be evaluated at an operating point. Here, one choose this point as the current value of *X*_4_, thereby always computing a term ${\alpha X}_{4}^{g}$ that is equivalent with *F*. The resulting system is

$\dot{X}_{1} =\alpha{X_{4}^{g} X}_{3}^{-0.5}- 0.5 X_{1}^{0.5}$ (S30a)

$\dot{X}_{2} =X_{1}- 5 X_{2}^{0.5}$ (S30b)

$\dot{X}_{3} =2 X_{2}- 3 X_{3}$ (S30c)

$\dot{X}_{4} =3 X_{3}- 12 X_{4}^{0.75}$ (S30d)

where

$g =(32 \frac{X_{4}^{4} \left( 4^{4}+ X_{4}^{4}- X_{4}^{3} \right)}{\left( 4^{4}+ X_{4}^{4} \right)^{2}} / ( 4+8 \frac{X_{4}^{4}}{4^{4}+ X_{4}^{4}} )$ (S31)

and

$\alpha= \left( 4+8 \frac{X_{4}^{4}}{4^{4}+ X_{4}^{4}} \right) X_{4}^{-g}$ (S32)

Note that $\alpha$ and *g* in this formulation are not constant, as it is typical in BST, but functions of *X*_4_ and thus of time.

**Supplements Section S5: Limit Cycles**

The Goodwin model [7, 8], expanded from three to four variables, has the form

$\dot{X}_{1} =k_{1} \frac{K^{n}}{K^{n}+ X_{4}^{n}} - k_{2} X_{1}$ (S33a)

$\dot{X}_{2} =k_{3}X_{1} - k_{4} X_{2}$ (S33b)

$\dot{X}_{3} =k_{5}X_{2} - k_{6} X_{3}$ (S33c)

$\dot{X}_{4} =k_{7}X_{3} - k_{8} X_{4}$ (S33d)

where the first equation does not adhere to the power-law format. To convert ("recast" [4]) the model, one proceeds as in the previous section and defines the auxiliary variable

$X_{5}=K^{n}+ X_{4}^{n}.$ (S34)

Its derivative is

$$\dot{X}_{5}=n X_{4}^{n-1} \dot{X}_{4}$$

$=n k_{7}X_{3} X_{4}^{n-1} - n k_{8} X_{4}^{n},$

$X_{5}\left( 0 \right)=K^{n}+ X_{4}^{n}\left( 0 \right).$ (S35)

Furthermore, the ODE for *X*_1_ becomes

$\dot{X}_{1} =k_{1} {K^{n}X}_{5}^{-1} - k_{2} X_{1}.$ (S36)

All equations are now in power-law format, and the dynamics of *X*_1_, …, *X*_4_ is exactly the same as in the original system.

For *n* = 4, reducing *X*_1_ or *X*_3_ leads to substantial approximation errors, in the case of *Y*_3_ essentially eliminating the oscillations (Fig. S1)


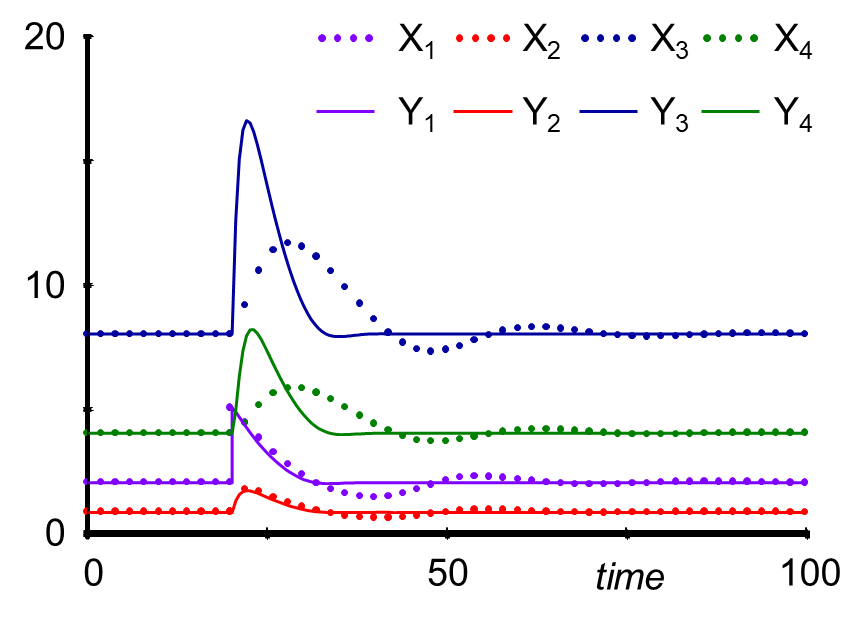


**Fig. S1**. Reduction of *X*_3_ essentially eliminates the oscillations of the system.

For *n* = 18, the reduction of some variables retains a limit cycle (see *Main Text*), but that is not always the case. If *X*_2_, *X*_3_, *X*_4_, or *X*_5_ are reduced, the limit cycle disappears, and the system displays damped oscillations instead (Fig. S2).


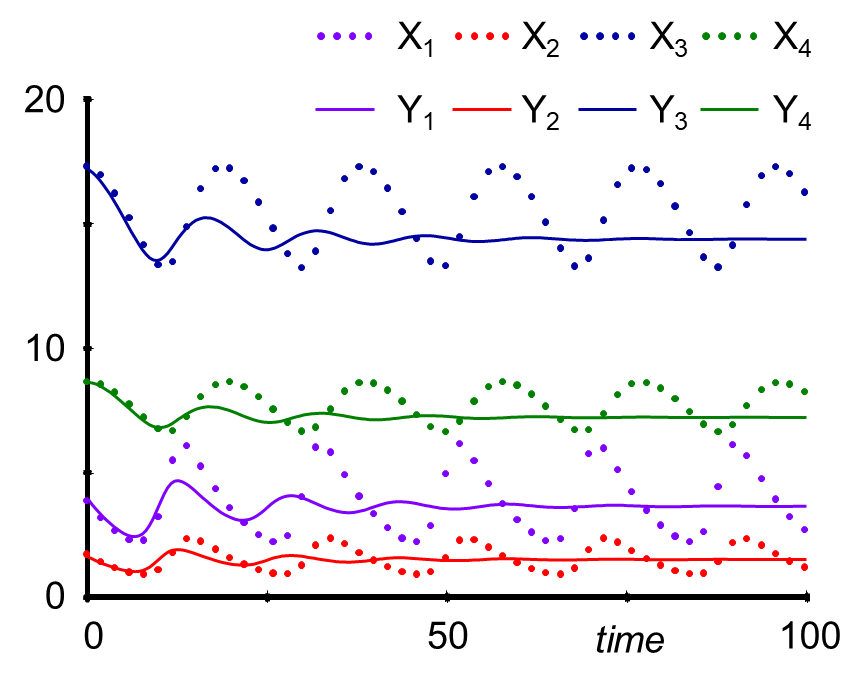


**Fig. S2**. Trajectories in response to the reduction of *X*_2_. The solution is initiated directly on the limit cycle of the original system, as this system does not have a stable steady state.

**Supplements Section S6:** **White-Rot Fungus Model**

The model of biomass production in the white-rot fungus *Phanerochaete chrysosporium* follows the work of Hormiga and colleagues [9]. The model is in the format of a mass action system, which is augmented with regulatory signals and a two-variable delay, yielding a GMA system. In accordance with the base structure of a mass action system, including regulation, we formulate the model as follows.

Gluc: $\dot{X}_{1} =V_{in\_Gluc} - V_{1} - V_{2}$

Biomass: $\dot{X}_{2} =V_{1} + V_{5} - V_{3}$

Dph: $\dot{X}_{3} =V_{in\_Dph} - V_{4}$

Mph: $\dot{X}_{4} ={2 V}_{4}- V_{5}$

Delay 1: $\dot{X}_{10} =K (X_{1}- X_{10})$

Lip: $\dot{X}_{5} =V_{6} - V_{7} + V_{8} - V_{9}$

Lip*: $\dot{X}_{6} =- V_{8} + V_{9}$

H_2_O_2_: $\dot{X}_{8} = V_{10}- V_{11}$

Ox: $\dot{X}_{9} =V_{12}- V_{13}$

Delay 2: $\dot{X}_{11} =K (X_{10}- X_{11})$

(S37)

*X*_7_ (O_2_ and H_2_O) and *X*_12_ (veratryl alcohol) are constant, independent variables. The fluxes are defined as follows:

*V_in_Gluc_* = 1

*V_in_Dph_* = 1

*V*_1_ = 0.3 *X*_11_

*V*_2_ = 0.1 *X*_1_

*V*_3_ = 0.3 *X*_2_

*V*_4_ = 0.4 *X*_3_ *X*_6_*^g^*^1^ X_12_*^g^*^2^

*V*_5_ = 0.5 *X*_4_

*V*_6_ = 0.1 *X*_1_*^g^*^3^ *X*_2_ *X*_12_*^g^*^2^

*V*_7_ = 0.1 *X*_5_

*V*_8_ = 0.2 *X*_6_ *X*_3_*^g^*^5^

*V*_9_ = 0.3 *X*_5_ *X*_8_*^g^*^4^

*V*_10_ = 0.2 *X*_7_ *X*_9_*^g^*^6^

*V*_11_ = 0.3 *X*_8_ *X*_5_*^g^*^7^

*V*_12_ = 0.1 *X*_1_*^g^*^3^ *X*_2_ *X*_12_^g2^

*V*_13_ = 0.4 *X*_9_

(S38)

If one replaces *X*_11_ with *X*_1_ in flux *V*_1_, the time delay is eliminated.

Hormiga and collaborators performed a formal parameter estimation for a situation without the external supply of substrate inputs [9]. Because we are here considering an *in vitro* system with constant inputs, their parameter values only provide coarse guidance. For simplicity, all activation parameters (*g*_1_, *g*_2_, *g*_4_, …, *g*_7_) are set equal to 0.5 and the inhibition parameter *g*_3_ to –1. Finally, we set the time delay parameter *K* = 0.2 and define the initial conditions as the steady-state values of the system:

*X*_1ss_ = 2.5

*X*_2 ss_ = 9.166667

*X*_3ss_ = 1.047034

*X*_4ss_ = 4

*X*_5ss_ = 4.490731

*X*_6ss_ = 3.800733

*X*_7ss_ = 1

*X*_8ss_ = 0.3333333

*X*_9ss_ = 1.122683

*X*_10ss_ = 2.5

*X*_11ss_ = 2.5

*X*_12ss_ = 1.5

(S39)

*Effects of reducing delays*

Reducing *X*_10_, *X*_11_, or both totally eliminates the oscillations caused by the time delay (Fig. S3).


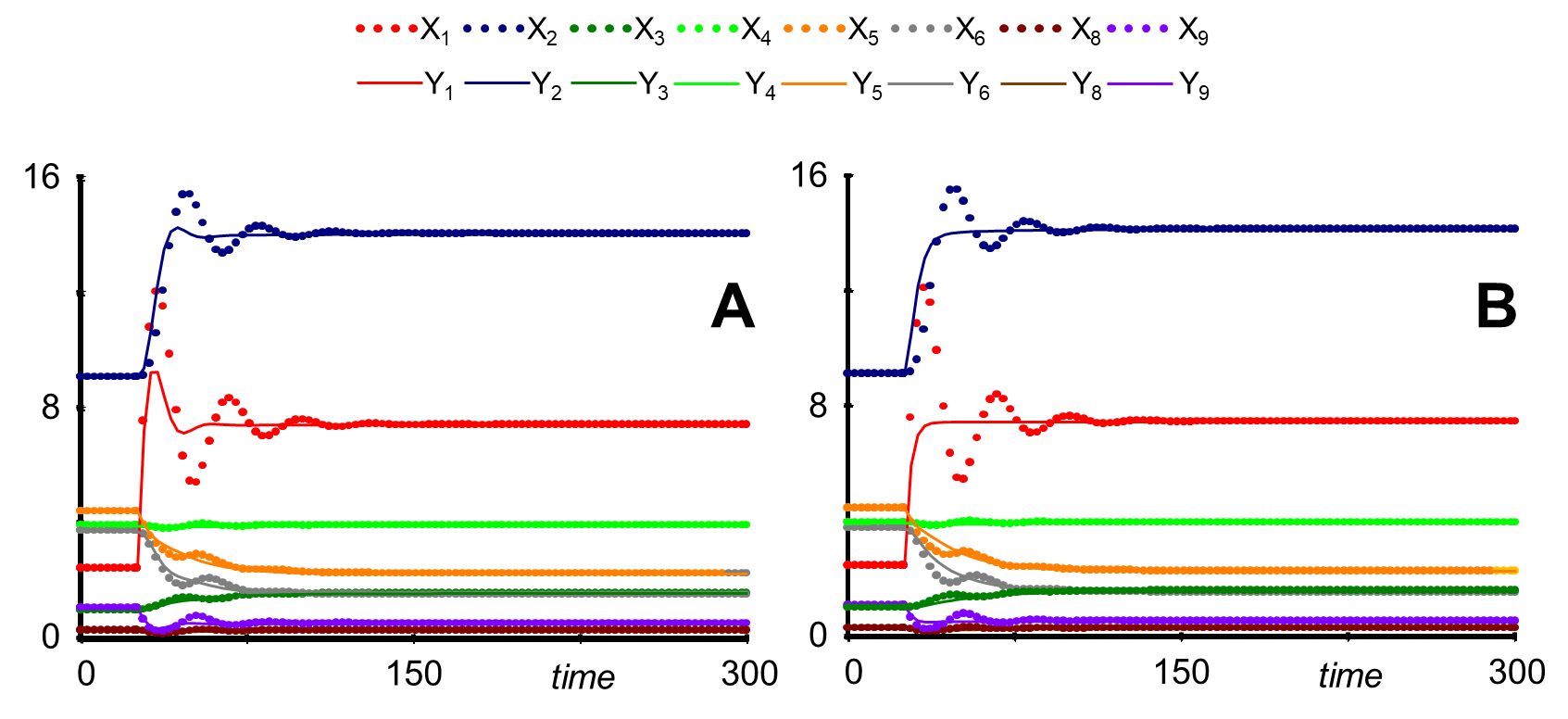


**Fig. S3: A**. Trajectories after reducing *X*_10_; reducing *X*_11_ results in trajectories that are visually the same.

**B**. Simultaneous reduction of *X*_10_ and *X*_11_. Note that *X*_7_ is constant and therefore not shown.

*Effects of reducing metabolite equations*

Reduction of *X*_3_ yields trajectories very close to those of the original model, except that *Y*_4_ is very slightly off (Fig. S4).


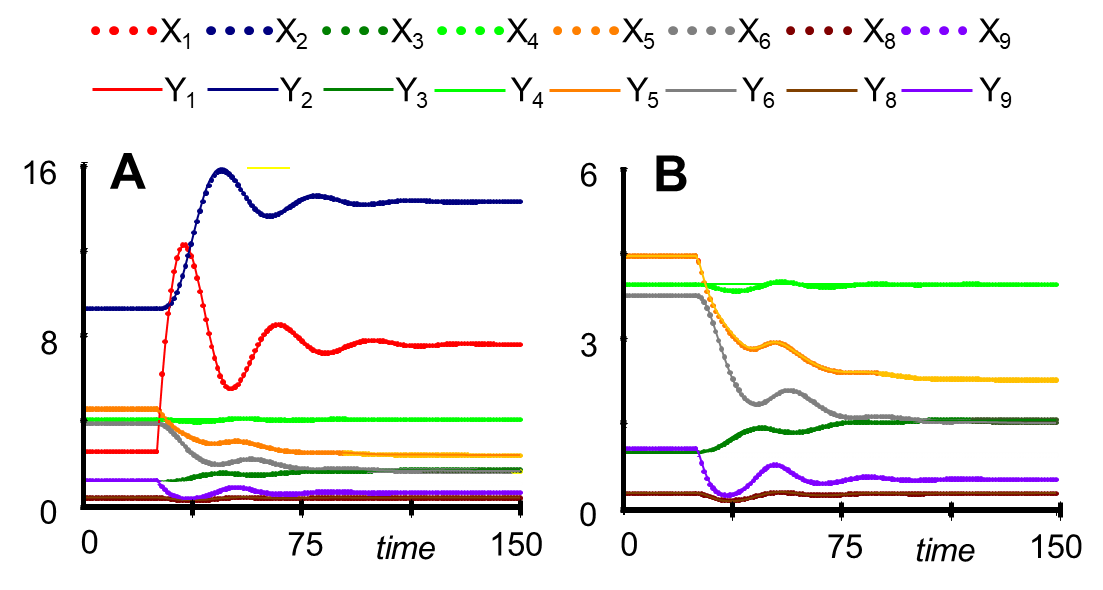


**Fig. S4.** Trajectories following reduction of *X*_3_. **A**. All variables. **B**. Variables of smaller magnitude (indices 3 - 9).

Reduction of *X*_4_ results in an essentially perfect match with the original (not shown). Reduction of *X*_5_ primarily affects *X*_3_ and *X*_5_ (Fig. S5). It also slows down the simulation (see later discussion).


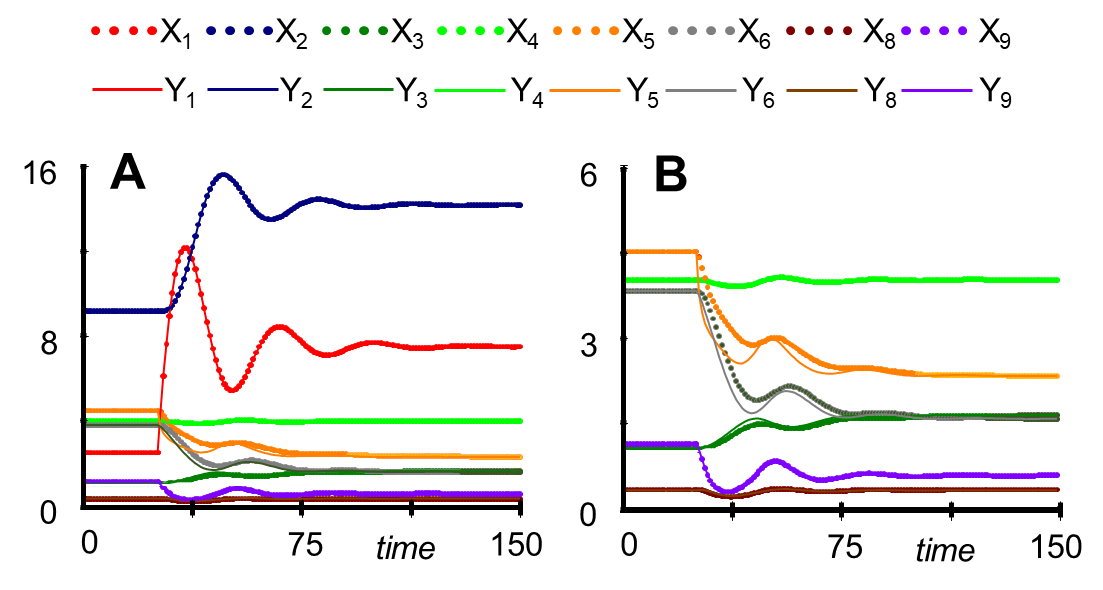


**Fig. S5**. Reducing *X*_5_ essentially leaves the trajectories of glucose (*Y*_1_) and biomass (*Y*_2_) unaltered.

It does affect *Y*_3_ and *Y*_5_. **A**. All variables. **B**. Variables of smaller magnitude (indices 3 - 9).

Reducing *X*_6_ results in rather substantial changes in oscillations, with *Y*_3_ - *Y*_6_ being affected most (Fig. S6).


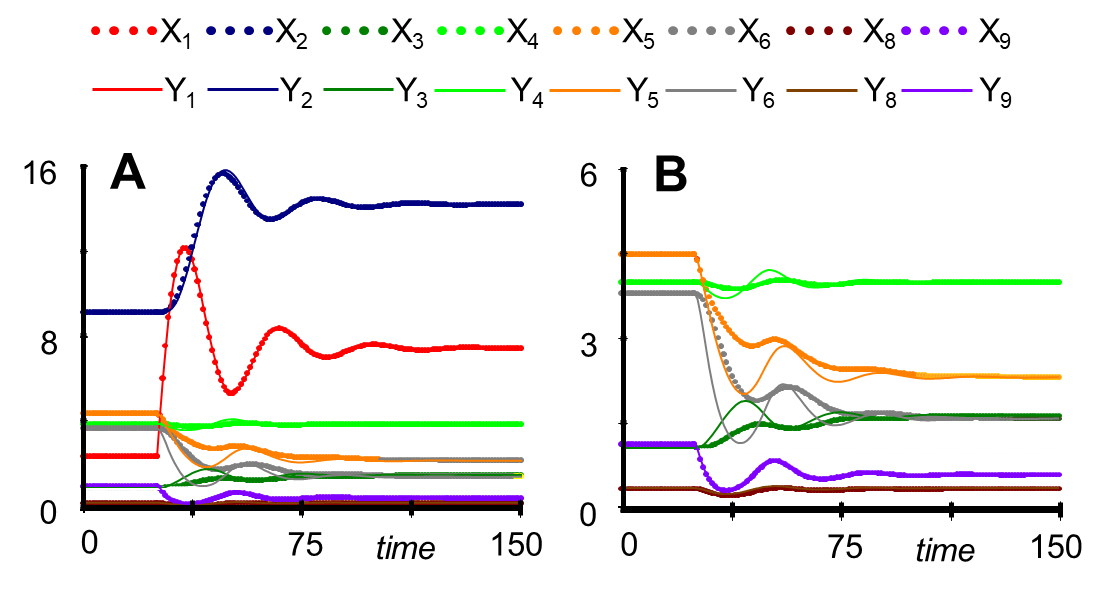


**Fig. S6**. Reduction of *X*_5_ mostly affects variables associated with the diphenol pathway (*Y*_3_ - *Y*_6_).

**A**. All variables. **B**. Variables of smaller magnitude (indices 3 - 9).

*X*_7_ is an independent variable that is not be reduced. Reduction of *X*_8_ yields essentially perfect results (Fig. S7).


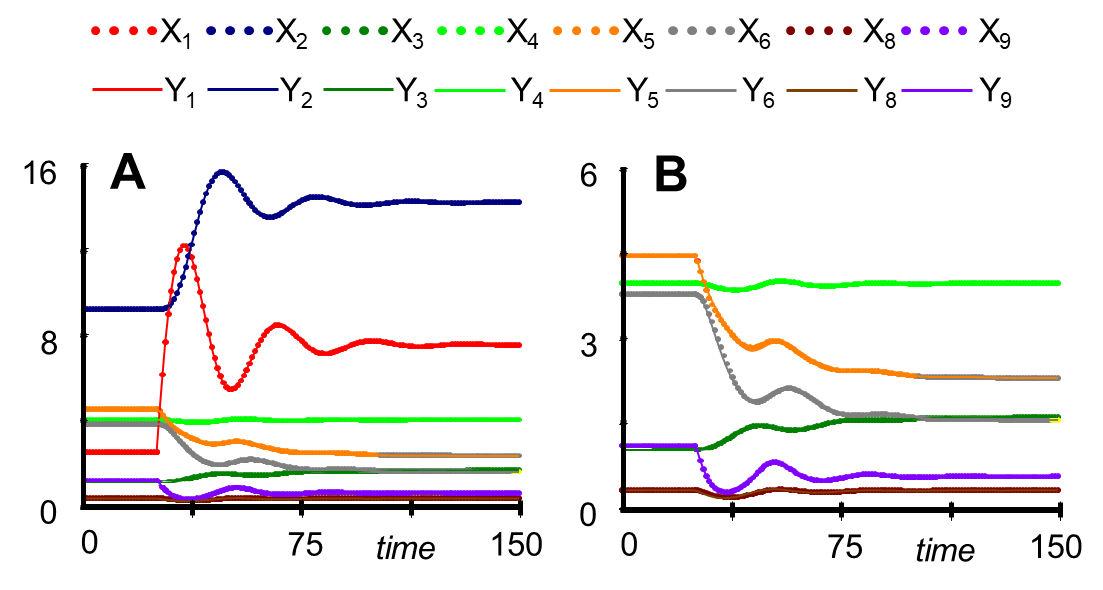


**Fig. S7**. Reduction of *X*_8_ yields essentially perfect results. **A**. All variables.

**B**. Variables of smaller magnitude (indices 3 - 9).

Reducing *X*_9_ does not affect the systems trajectories much, with the exception of *Y*_9_ itself, which oscillates slightly faster. There are also minute deviations in *Y*_5_ and *Y*_6_ (Fig. S8).


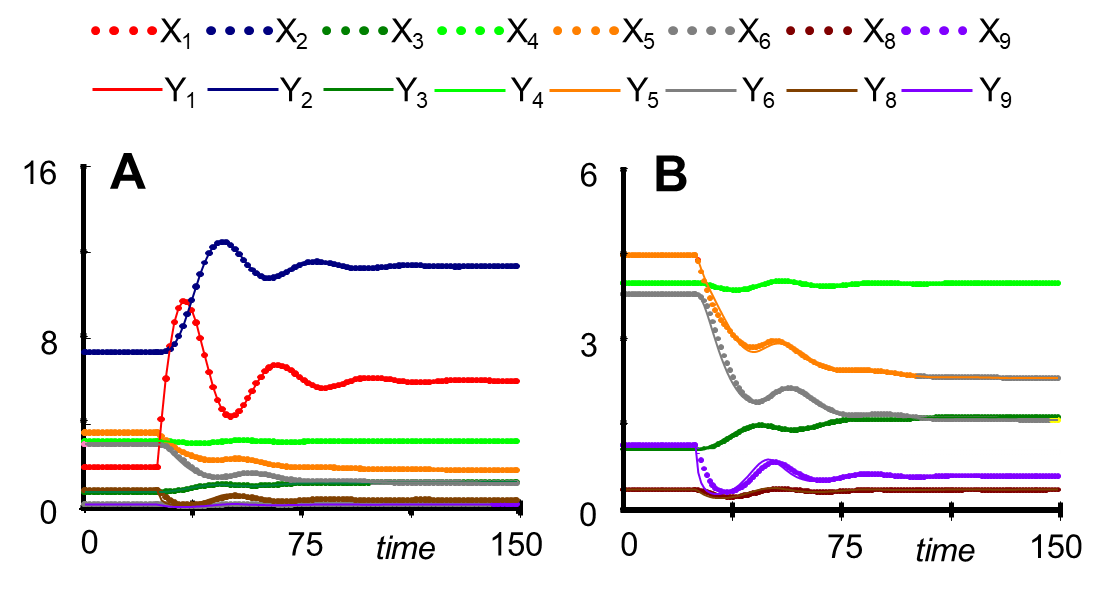


**Fig. S8.** Reduction of *X*_9_ yields very good results. **A**. All variables.

**B**. Variables of smaller magnitude (indices 3 - 9).

*Simultaneous reduction of several variables*

For LV- and S-systems, reduction of an ODE results in the variable expressed, respectively, as a linear or a power-law term containing other variables. This term can be substituted directly into all remaining equations, thereby eliminating the variable. In cases of other formats, including GMA systems, the process is sometimes not quite as clean.

As an example, consider the simultaneous reduction of *X*_5_ and *X*_6_. The reduced equations are

$Y_{5}=\left( 0.1 Y_{1}^{g_{3}} Y_{2}Y_{12}^{g_{2}}+ 0.2 Y_{6}Y_{3}^{g_{5}} \right) / (0.1+0.3 Y_{8}^{g_{4}})$ (S40)

and

$Y_{6}=0.3 Y_{5}Y_{8}^{g_{4}} / (0.2 Y_{3}^{g_{5}}).$ (S41)

They show that *Y*_5_ depends on *Y*_6_ and *vice versa*. This mutual dependence can pose problems for the solution algorithm, starting with the initial value. The most effective solution to the issue is to manipulate the equations algebraically so that the mutual dependence is eliminated.

For the given case, it is useful to convert *Y*_5_ into a sum of power-law terms, by representing the denominator $F=0.1+0.3 Y_{8}^{g_{4}}$ as a power-law of the type ${\alpha Y}_{8}^{g}$. According to the principles of the power-law approximation, the kinetic order *g* is given as $g= \frac{dF}{dY_{8}}\frac{Y_{8}}{F}$ and *α* as $F Y_{8}^{-g};$ both are to be evaluated at an operating point. As before, we choose this point as the current value of *Y*_8_, thereby computing a term ${\alpha Y}_{8}^{g}$that is always equivalent with *F*, where $\alpha$ and *g* are impicit functions of time via the involvement of *Y*_8_. Thus, the definitions

$g=0.3 g_{4}Y_{8}^{g_{4}} / (0.1+0.3 Y_{8}^{g_{4}})$ (S42)

$\alpha= (0.1+0.3 Y_{8}^{g_{4}})Y_{8}^{-g}$ (S43)

lead to a PL formulation that is equivalent to the Eq. (S40):

$Y_{5}=\left( 0.1 Y_{1}^{g_{3}} Y_{2}Y_{12}^{g_{2}}+ 0.2 Y_{6}Y_{3}^{g_{5}} \right) / (\alpha Y_{8}^{g})$ (S44)

Secondly, we solve *Y*_5_ for *Y*_6_. Simple algebraic manipulation of (S44) yields

$Y_{5} \cdot\alpha Y_{8}^{g}- 0.1 Y_{1}^{g_{3}} Y_{2}Y_{12}^{g_{2}}=0.2 Y_{6}Y_{3}^{g_{5}}$ (S45)

$Y_{6}=\left( Y_{5} \cdot\alpha Y_{8}^{g}- 0.1 Y_{1}^{g_{3}} Y_{2}Y_{12}^{g_{2}} \right)/ (0.2Y_{3}^{g_{5}})$ (S46)

The expression (S46) is now equated with the reduced equation for *Y*_6_ in (S41). As a result, *Y*_6_ is eliminated, and the solution for *Y*_5_ is

$Y_{5}= 0.1 Y_{1}^{g_{3}} Y_{2}Y_{12}^{g_{2}} / \left( \alpha Y_{8}^{g}-0.3 Y_{8}^{g_{4}} \right)$ (S47)

The solution algorithm can now first evaluate *Y*_5_ and subsequently *Y*_6_ via (S41).

*Results of simultaneous reductions*

Simultaneous reduction of *X*_5_ and *X*_6_ leads to slight deviations in the trajectory of biomass but substantial changes with respect to most variables leading to biomass production from diphenol (Fig. S9).


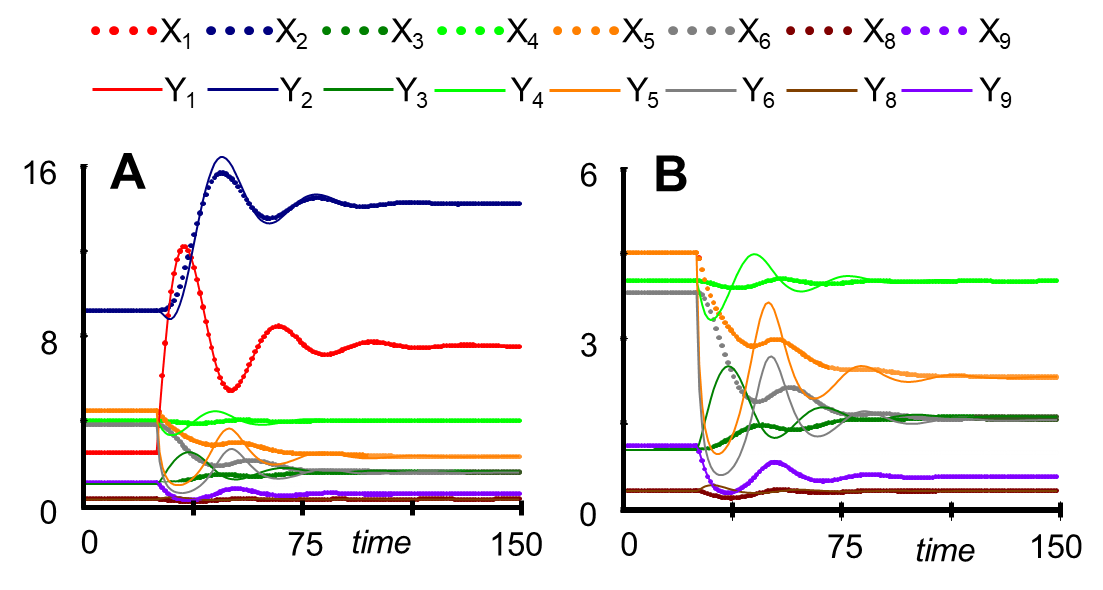


**Fig. S9.** Reduction of *X*_5_ and *X*_6_ causes slight changes in biomass dynamics and substantial alterations

of the trajectories of *Y*_3_ – *Y*_8._ **A**. All variables. **B**. Variables of smaller magnitude (indices 3 - 9).

If only biomass is of interest, *X*_3_-*X*_9_ can be reduced with relative accuracy (see *Main Text*). Reducing *X*_10_ and *X*_11_ as well eliminates overshoots and undershoots and speeds up the dynamics of all equations. In fact, the entire dynamics in this case is quite different (Fig S10).


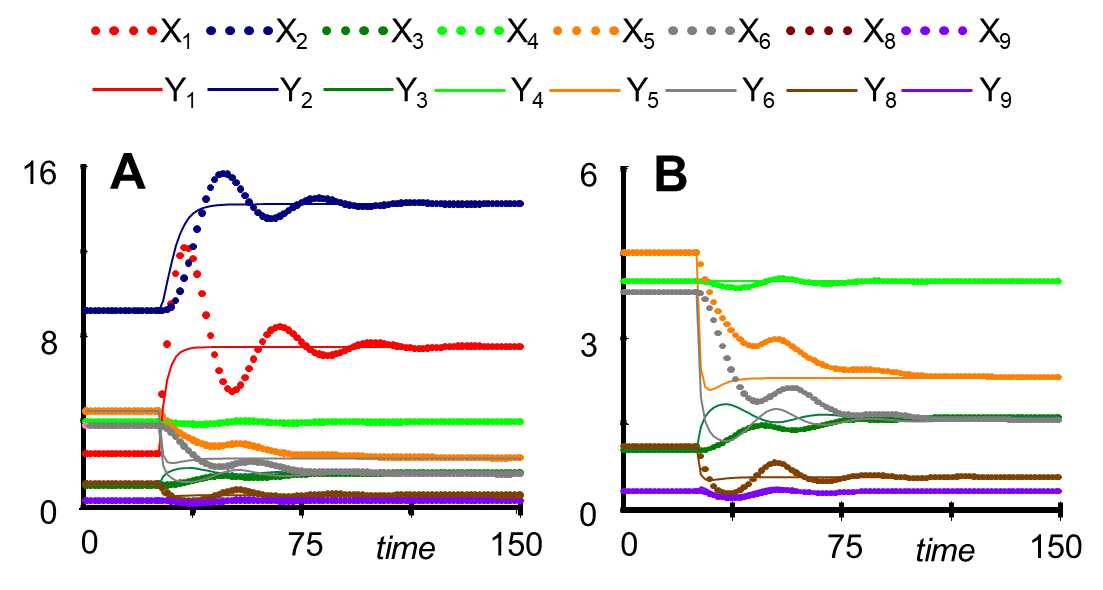


**Fig. S10.** Reduction of *X*_3_ – *X*_11_ leads to a dynamics that is very different from that of the original model.

**A**. All variables. **B**. Variables of smaller magnitude (indices 3 - 9).

**Supplements Section S7: Competition Among Bacterial Populations**

Davis and collaborators [10] analyzed data published by Piccardi *et al.* [11] with a four-variable Lotka-Volterra (LV) model. The data were experimentally obtained for four bacterial species coexisting in metal working fluids (see *Main Text*). This system is interesting because some components of these fluids are used as growth substrates, while some are toxic. The generic format of the model is

$$\dot{X}_{i}=a_{i}X_{i}+ \sum_{j=1}^{4} b_{ij}{X_{i}X}_{j}, i = 1, \ldots, 4. (S48)$$

The parameter values and initial conditions are presented in Table S2.

As explained in the *Main Text*, one of the species in Piccardi's experiment eventually disappeared, thereby creating a situation without non-trivial steady state (Table S2). However, changing just one growth rate (*a*_2_) by 20%, the model achieves a stable steady state. In the *Main Text*, this implementation was used. The following contains comments on reductions of the original model of Davis [10].

**Table S2.** Parameter settings of a model of four competing bacterial species.

| **Variable** | **Species Name** | **Linear Term** | **Interaction Terms** | **Initial Value** |
| --- | --- | --- | --- | --- |
| *X*_1_ | *A. tumefaciens* | *a*_1_ = 0.0196 | *b*_11_ = -1.82×10^-10^  *b*_12_ = -5.51×10^-12^  *b*_13_ = -2.51×10^-11^  *b*_14_ = 2.18×10^-11^ | 1.0×10^7^ |
| *X*_2_ | *C. testosteroni* | *Original:*  *a*_2_ = 0.0744  *Steady-state model:*  *a*_2_ = 0.08928 | *b*_21_ = -7.94×10^-10^  *b*_22_ = -2.44×10^-11^  *b*_23_ = 1.79×10^-11^  *b*_24_ = 3.47×10^-11^ | 0.8×10^7^ |
| *X*_3_ | *M. saperdae* | *a*_3_ = 0.00542 | *b*_31_ = 2.46×10^-10^  *b*_32_ = -1.16×10^-11^  *b*_33_ = -3.54×10^-10^  *b*_34_ = 7.28×10^-11^ | 4.0×10^7^ |
| *X*_4_ | *O. anthropi* | *a*_4_ = 0.0515 | *b*_41_ = -4.88×10^-11^  *b*_42_ = 6.01×10^-11^  *b*_43_ = 6.22×10^-11^  *b*_44_ = -2.06×10^-10^ | 0.8×10^7^ |

At the beginning of the growth phase, the slopes of variables *X*_1_, *X*_2_, and *X*_4_ are steep and thus quite distant from 0 (Fig. S11). This fact causes problems for most reductions. Attempting to reduce *X*_1_ or *X*_2_ yields unacceptable results (Fig. S12). In the latter case, even the initial conditions are far off and *Y*_2_ precipitously crashes before the final time of the original simulation.


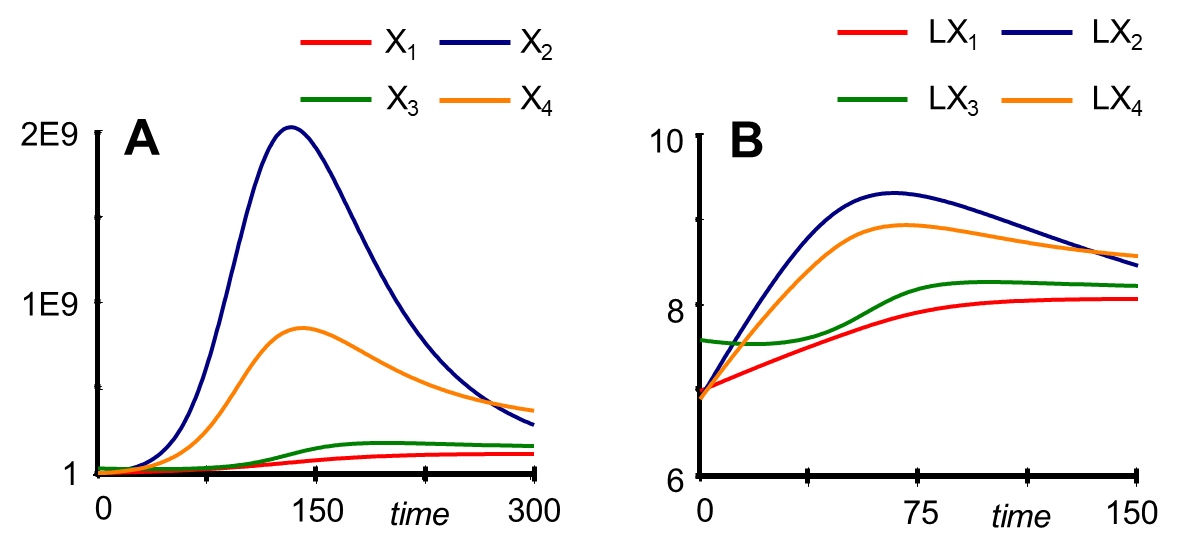


**Fig. S11**. Trajectories of all four species in Davis’ model [10].

**A**: Population abundances in Cartesian coordinates. **B**: Logarithms of abundances.


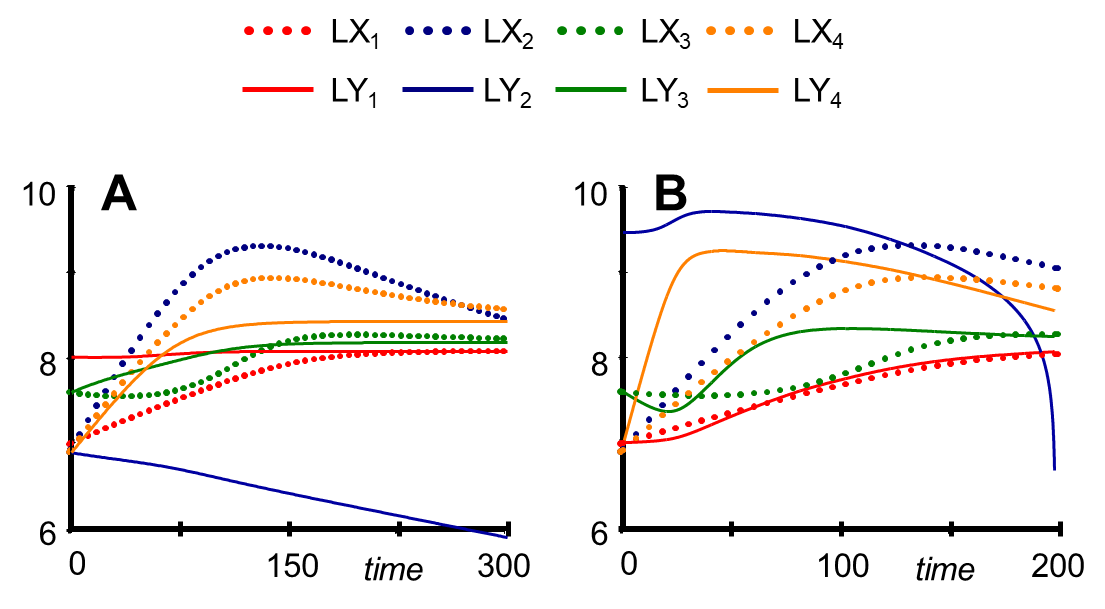


**Fig. S12**. Trajectories following reduction of *X*_1_ (**A**) or *X*_2_ (**B**) in logarithmic values.

Reduction of *X*_3_ yields very different results, possibly because its slope at the beginning of the simulation is quite flat (Fig. S13A). Reduction of *X*_4_ leads to trajectories that are acceptable for *Y*_1_ and *Y*_2_ although not for *Y*_3_ and *Y*_4_ (Fig. S13B). Reducing *X*_3_ and *X*_4_ simultaneously results in trajectories similar to Fig. S13B, with the trajectory of *Y*_3_ further shifted up (not shown).


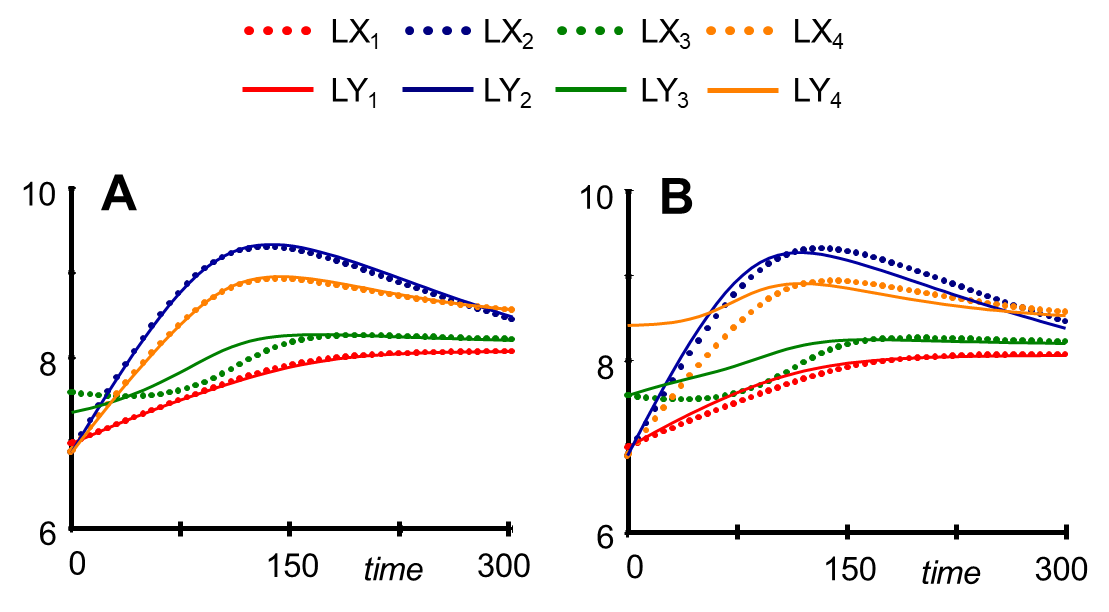


**Fig. S13**. Trajectories following reduction of *X*_3_ (**A**) or *X*_4_ (**B**) in logarithmic values.

After about 150 time units into the experiment, *X*_2_ and *X*_4_ are reaching their peaks, with slopes that are much closer to 0 than in the beginning of the experiment. If the simulation starts close to the high point of population abundance, with (*X*_1_(*t* = 150), … *X*_4_(*t* = 150)) = (8.415028×10^7^, 1.895268×10^9^, 1.55557×10^8^, 8.426012×10^8^), the reduction of *X*_1_ is much improved, although still noticeably different from the original. A reduction of *X*_2_ again fails, while reductions of *X*_3_ and *X*_4_ yield close to perfect results (Fig. S14). Taken together, *X*_2_ cannot be reduced successfully, possibly identifying it as a key variable. However, caution is needed with this interpretation, as the system does not have a non-trivial steady state.


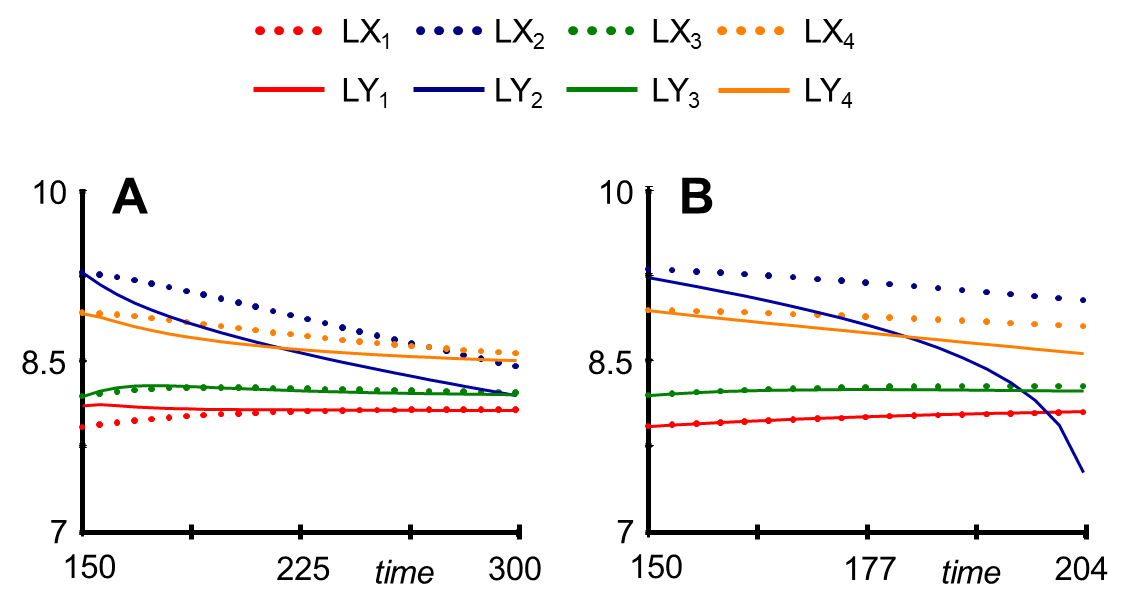


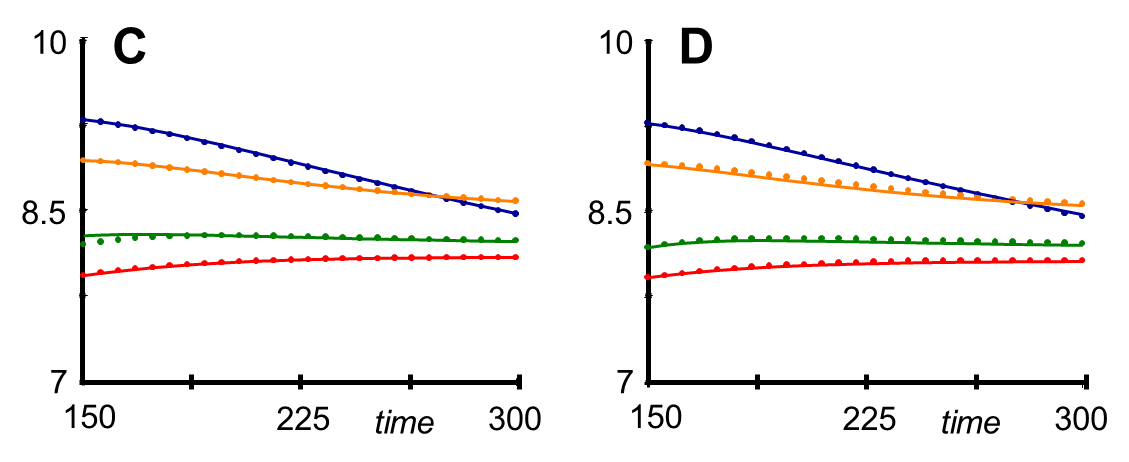


**Fig. S14**. Trajectories following reduction of *X*_1_ (**A**), *X*_2_ (**B**), *X*_3_ (**C**), or *X*_4_ (**D**),

when simulations are started at *t* = 150.

**References**

1. Curien, G., et al., *Understanding the regulation of aspartate metabolism using a model based on measured kinetic parameters.* Mol Syst Biol, 2009. **5**: p. 271.

2. Iwata, M., F. Shiraishi, and E.O. Voit, *Coarse but efficient identification of metabolic pathway systems.* Int. J. Syst. Biol., 2013. **4**(1): p. 57-72.

3. Hill, A.V., *Possible effects of the aggregation of the molecules of haemoglobin on its dissociation curves.* J. Physiol, 1910. **40**: p. iv – viii.

4. Savageau, M.A. and E.O. Voit, *Recasting nonlinear differential equations as S-systems: A canonical nonlinear form.* Mathem Biosci, 1987. **87**: p. 83-115.

5. Voit, E.O. and M.A. Savageau, *Equivalence between S-Systems and Volterra Systems.* Mathematical Biosciences, 1986. **78**(1): p. 47-55.

6. Voit, E.O., *Biochemical Systems Theory: A review.* Int. Scholarly Res. Network (ISRN – Biomathematics), 2013. **Article 897658**: p. 1-53.

7. Goodwin, B.C., *Oscillatory behavior in enzymatic control processes.* Adv Enzyme Regul, 1965. **3**: p. 425-38.

8. Gonze, D. and W. Abou-Jaoude, *The Goodwin model: behind the Hill function.* PLoS One, 2013. **8**(8): p. e69573.

9. Hormiga, J.A., et al., *Growth and ligninolytic system production dynamics of the Phanerochaete chrysosporium fungus: A modelling and optimization approach.* Journal of Biotechnology, 2008. **137**(1): p. 50-58.

10. Davis, J.D., et al., *Methods of quantifying interactions among populations using Lotka-Volterra models.* Front. Systems Biology, 2022. **2**.

11. Piccardi, P., B. Vessman, and S. Mitri, *Toxicity drives facilitation between 4 bacterial species.* 2019. **116**(32): p. 15979-15984.
